# SIRT2 suppresses aging-associated cGAS activation and protects aged mice from severe COVID-19

**DOI:** 10.1016/j.celrep.2025.115562

**Published:** 2025-04-11

**Authors:** Marine Barthez, Biyun Xue, Jian Zheng, Yifei Wang, Zehan Song, Wei-Chieh Mu, Chih-ling Wang, Jiayue Guo, Fanghan Yang, Yuze Ma, Xuetong Wei, Chengjin Ye, Nicholas Sims, Luis Martinez-Sobrido, Stanley Perlman, Danica Chen

**Affiliations:** 1 Department of Nutritional Sciences and Toxicology, University of California, Berkeley, Berkeley, CA 94720, USA; 2 Department of Microbiology and Immunology, University of Iowa, Iowa City, IA, USA; 3 Department of Microbiology and Immunology, Center for Predictive Medicine, University of Louisville, Louisville, KY, USA; 4 Metabolic Biology Graduate Program, University of California, Berkeley, Berkeley, CA 94720, USA; 5 Endocrinology Graduate Program, University of California, Berkeley, Berkeley, CA 94720, USA; 6 Texas Biomedical Research Institute, San Antonio, TX, USA; 7 Department of Pediatrics, University of Iowa, Iowa City, IA, USA; 8 These authors contributed equally; 9 Lead contact

## Abstract

Aging-associated vulnerability to coronavirus disease 2019 (COVID-19) remains poorly understood. Here, we show that severe acute respiratory syndrome coronavirus 2 (SARS-CoV-2)-infected aged mice lacking SIRT2, a cytosolic NAD^+^-dependent deacetylase, develop more severe disease and show increased mortality, while treatment with an NAD^+^ booster, 78c, protects aged mice from lethal infection. Mechanistically, we demonstrate that SIRT2 modulates the acetylation of cyclic GMP-AMP synthase (cGAS), an immune sensor for cytosolic DNA, and suppresses aging-associated cGAS activation and inflammation. Furthermore, we show that SARS-CoV-2 infection-induced inflammation is mediated at least in part by ORF3a, which triggers mtDNA release and cGAS activation. Collectively, our study reveals a molecular basis for aging-associated susceptibility to COVID-19 and suggests therapeutic approaches to protect aged populations from severe SARS-CoV-2 infection.

## In brief

Barthez et al. show that SIRT2 and NAD^+^ boosting protect against aging-associated severe COVID-19. SARS-CoV-2-induced inflammation is mediated by ORF3a, which triggers mtDNA release and cGAS activation. SIRT2 deacetylates cGAS and suppresses aging-associated cGAS activation and inflammation. This study has implications for protecting aged populations from severe SARS-CoV-2 infection.

## Graphical Abstract

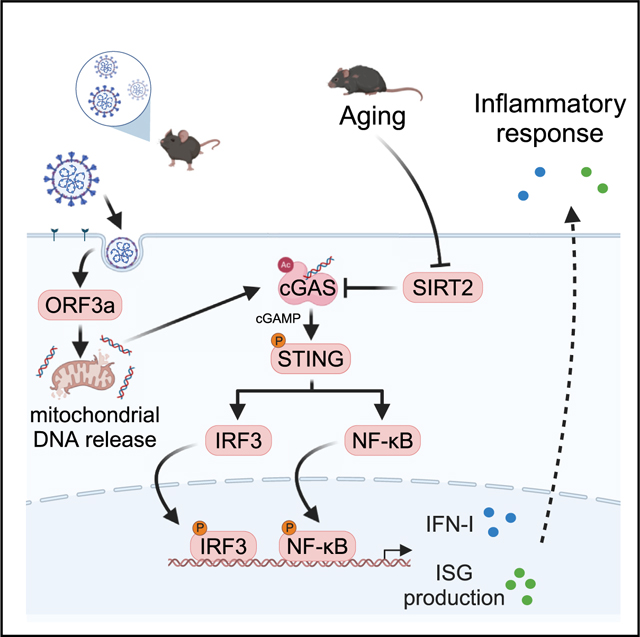

## INTRODUCTION

Severe acute respiratory syndrome coronavirus 2 (SARS-CoV-2) infection causes COVID-19, which is characterized by damage to the respiratory tract and, possibly, multi-organ failure and death.^1,2^ The COVID-19 pandemic has caused more than 776 million cases and 7.07 million confirmed deaths as of October 2024 according to the World Health Organization. COVID-19 disproportionally impacts aged populations, and aged compared to younger individuals have up to a 10,000-fold greater risk of mortality.^3–6^ Vaccines against SARS-CoV-2 are, in general, highly effective. However, vaccines are less immunogenic in aged individuals.^7–10^ Furthermore, the emergence of new variants that evade the protection of vaccines represents a challenge, and COVID-19 remains an ongoing global health problem.^11^ In September 2024, there were about 15,000 hospitalizations weekly in the United States due to COVID-19, pointing to the need for anti-SARS-CoV-2 therapies, especially for aged populations.

Inflammation is essential for clearing infection and repairing tissue damage. However, aging leads to chronic low-grade inflammation that is linked to aging-associated conditions and diseases.^12,13^ Aging-associated inflammation is mediated by immune cells and senescent cells.^14,15^ In addition to accumulation of cellular stress and damage, which trigger prolonged inflammation during aging,^13,16^ emerging evidence suggests that immune responses become aberrantly regulated with aging, resulting in reduced ability to suppress inflammation and return to homeostasis.^17^ Because COVID-19 results in part from aggressive inflammatory responses, aging-associated inflammation is likely to contribute to increased vulnerability of aged individuals to COVID-19 and may be targeted to alleviate COVID-19.

Mice cannot be infected with ancestral strains of SARS-CoV-2, but we and others have modified mice or the virus to enable mouse infection.^18–28^ In one instance, we inserted a single mutation into SARS-CoV-2, which facilitated murine infection. We then serially passaged virus through mouse lungs 30 times to enhance mouse adaptation and disease severity, resulting in a virus that caused lethal disease (SARS2-N501Y_MA30_).^27^ C57Bl/6 mice infected with SARS2-N501Y_MA30_ show age-dependent differences in mortality and morbidity.^27^

In this study, using SARS2-N501Y_MA30_-infected mice, we demonstrate that SIRT2 and NAD^+^ boosting protect aged mice from severe disease and lethal infection. Mechanistically, we show that SARS-CoV-2 infection induces inflammation resulting from ORF3a-induced mtDNA release and cyclic GMP-AMP synthase (cGAS) activation and that SIRT2 suppresses aging-associated cGAS activation and inflammation. Our findings support the notion that age-associated inflammation is a determining factor for increased vulnerability of aged individuals to SARS-CoV-2 infection and provide a basis for using NAD^+^ boosters to treat aged individuals for COVID-19.

## RESULTS

### SIRT2 protects aged mice from lethal SARS-CoV-2 infection

**Figure 1.**
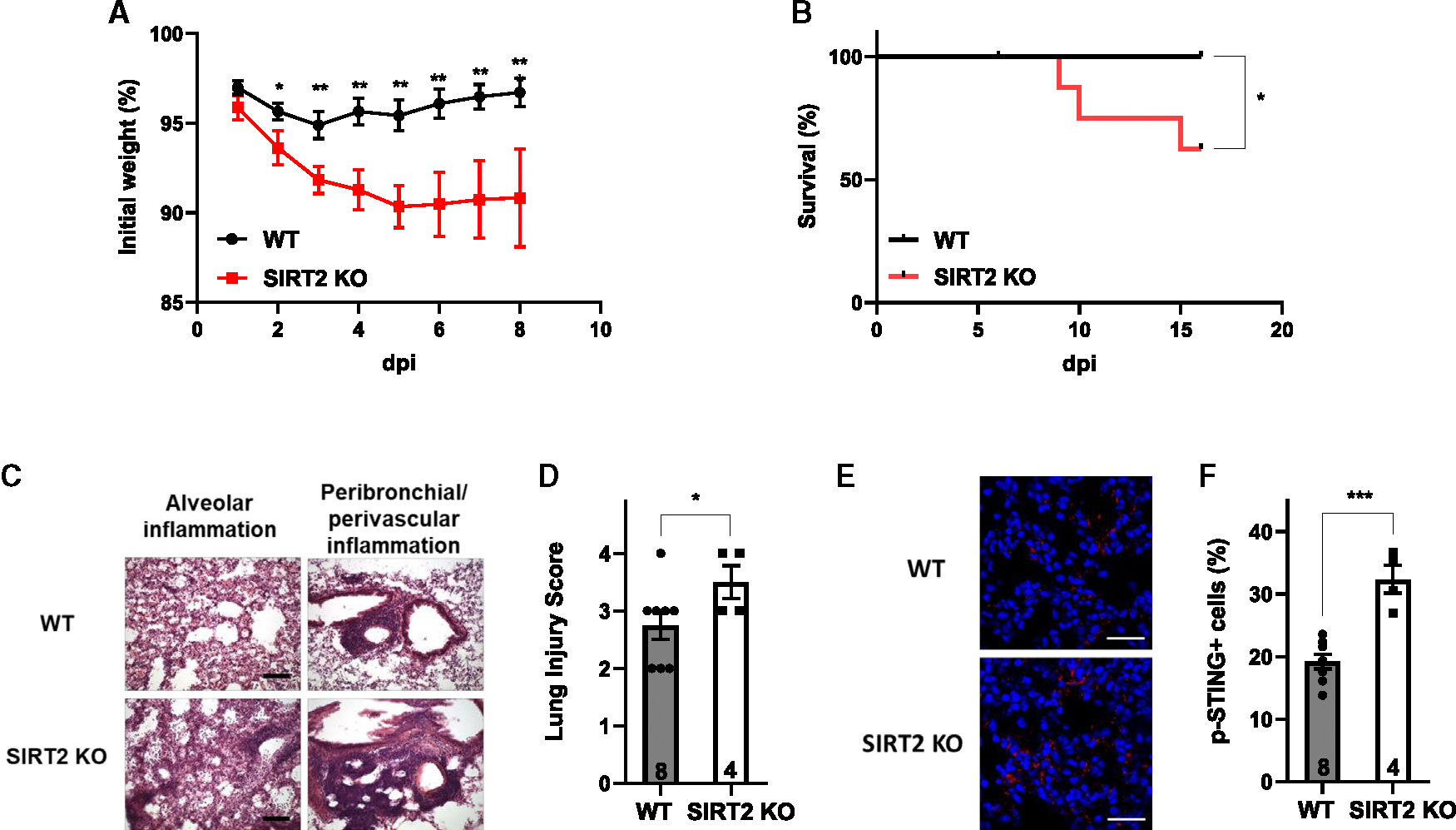
SIRT2 protects aged mice from lethal SARS-CoV-2 infection Aged (18–24 months old) WT and SIRT2 KO mice were infected with 1,000 PFUs of SARS2-N501Y_MA30_. *n* = 3 independent experiments. (A) Body weight. *n* = 14 WT and 8 SIRT KO mice/group. (B) Survival curve. *n* = 14 WT and 8 SIRT KO mice/group. (C and D) H&E staining (C) and quantification (D) of lung sections. Scale bar: 100 μm *n* = 8 WT and 4 SIRT KO mice/group, 10–15 images examined from 3 slides/mouse. (E and F) Immunostaining for phosphorylation of STING (E) and quantification (F) of lung sections. Scale bar: 30 μm *n* = 8 WT and 4 SIRT KO mice/group, 5 images examined from 3 slides/mouse. Data are mean ± SEM. *p < 0.05, **p < 0.01, ***p < 0.001. See also Figures S1 and S2.

SIRT2 is a regulator of aging-associated conditions. SIRT2 knockout (KO) mice do not have noticeable defects at a young age but show exacerbated aging-associated conditions at old age.^17,29^ To determine whether SIRT2 protects aged mice from severe SARS-CoV-2 infection, we infected old (18–24 months old) wild-type (WT) and SIRT2 KO mice with 1,000 plaque-forming units (PFUs) of SARS2-N501Y_MA30_, a mouse-adapted SARS-CoV-2 (Figure S1A).^27^ Aged SIRT2 KO mice lost more weight upon infection (Figure 1A). While no aged WT mice died after infection with this virus dose, 37.5% of aged SIRT2 KO mice succumbed (Figure 1B). Pathological changes (Figures 1C and 1D) and myeloid cell infiltration (Figures S1B–S1E) in the lungs were more severe in aged SIRT2 KO mice. In contrast, SARS-CoV-2 infection of 10-month-old WT and SIRT2 KO mice resulted in similar weight loss and survival rate (Figure S2).

While rapid induction of type I interferons (IFNs) limits virus propagation, sustained induction of type I IFNs in the late phase of SARS-CoV-2 infection is associated with aggressive inflammation and poor clinical outcomes.^2,30–40^ The cGAS-stimulator of IFN genes (STING) pathway is upregulated in severe COVID-19, driving type I IFN immunopathology.^30^ Phosphorylation of STING was increased in the lungs of aged SIRT2 KO mice upon viral infection (Figures 1E and 1F), consistent with a role of SIRT2 in suppressing cGAS activation. These results suggest that SIRT2 protects aged mice from severe disease by suppressing cGAS activation.

### NAD^+^ boosting protects aged mice from lethal SARS-CoV-2 infection

**Figure 2.**
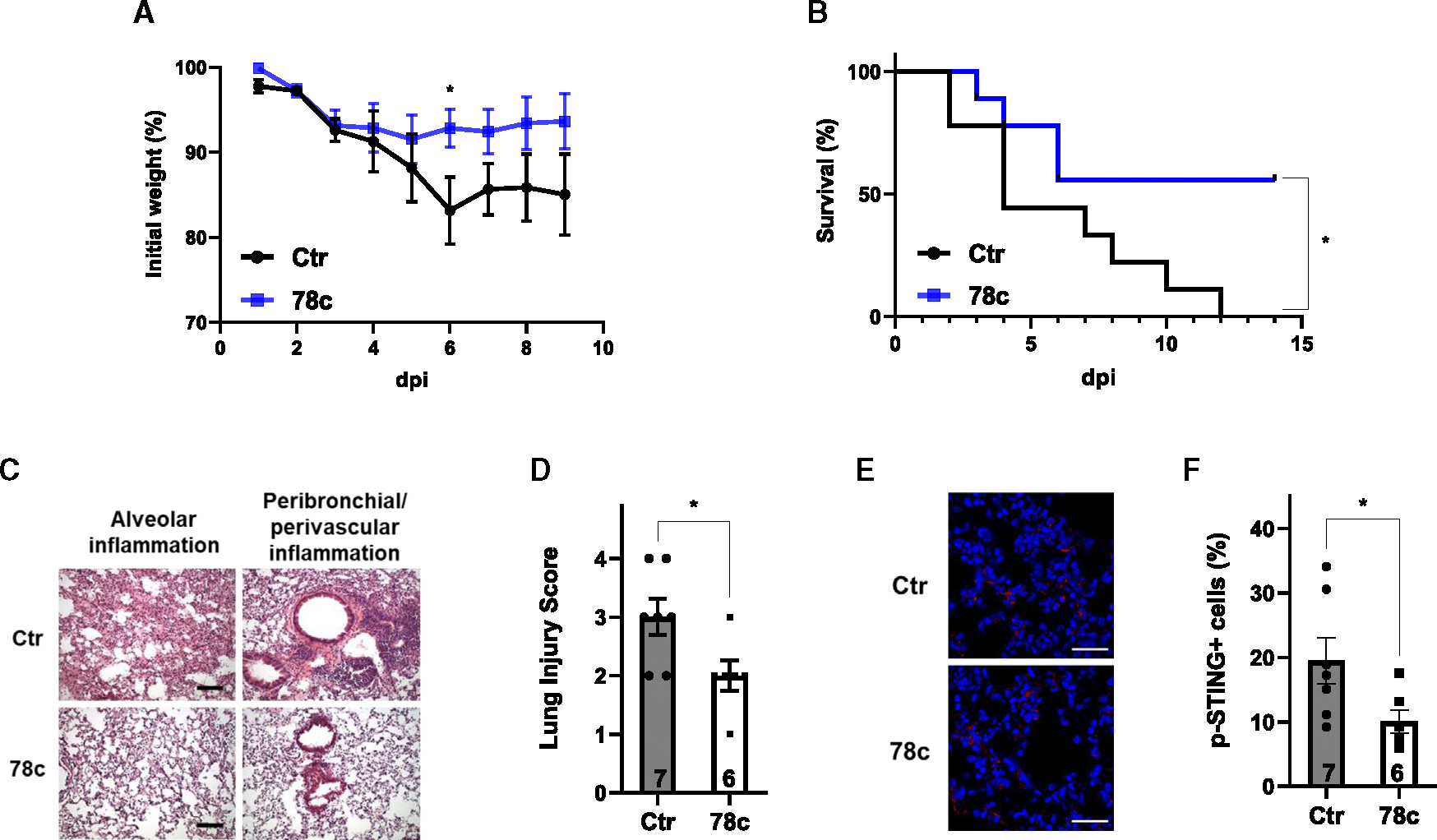
NAD^+^ boosting protects aged mice from lethal SARS-CoV-2 infection Aged (24 months old) WT mice were infected with 5,000 PFUs of SARS2-N501Y_MA30_ followed by treatment with or without 78c 1 dpi. *n* = 2 independent experiments. (A) Body weight. *n* = 9 mice/group. (B) Survival curve. *n* = 9 mice/group. (C and D) H&E staining (C) and quantification (D) of lung sections. Scale bar: 100 μm *n* = 7,6 mice/group, 10–15 images examined from 3 slides/mouse. (E and F) Immunostaining for phosphorylation of STING (E) and quantification (F) of lung sections. Scale bar: 30 μm *n* = 7,6 mice/group, 5 images examined from 3 slides/mouse. Data are mean ± SEM. *p < 0.05. See also Figure S3.

NAD^+^ boosters are emerging as attractive means to activate sirtuins, which possess NAD^+^-dependent deacetylase activity.^41–43^ We next administered 78c, an NAD^+^ booster,^44^ to 24-month-old C57BL/6 mice twice daily by intraperitoneal injection for 13 days starting 1 day post infection (dpi) with 5,000 PFUs of SARS2-N501Y_MA30_ (Figure S3A). A lethal dose of virus was used in these experiments to enable assessment of 78c protective efficacy. 78c treatment reduced body weight loss (Figure 2A) and decreased mortality from 100% to 44% (Figure 2B). Drug treatment resulted in diminished pathological changes in the lungs (Figures 2C and 2D) and reduced myeloid cell infiltration in the lungs (Figures S3B–S3E). Phosphorylation of STING was reduced in the lungs of mice treated with 78c (Figures 2E and 2F), consistent with activation of SIRT2 and suppression of cGAS signaling through NAD^+^ boosting.

### SIRT2 deacetylates cGAS to suppress cGAS activity

**Figure 3.**
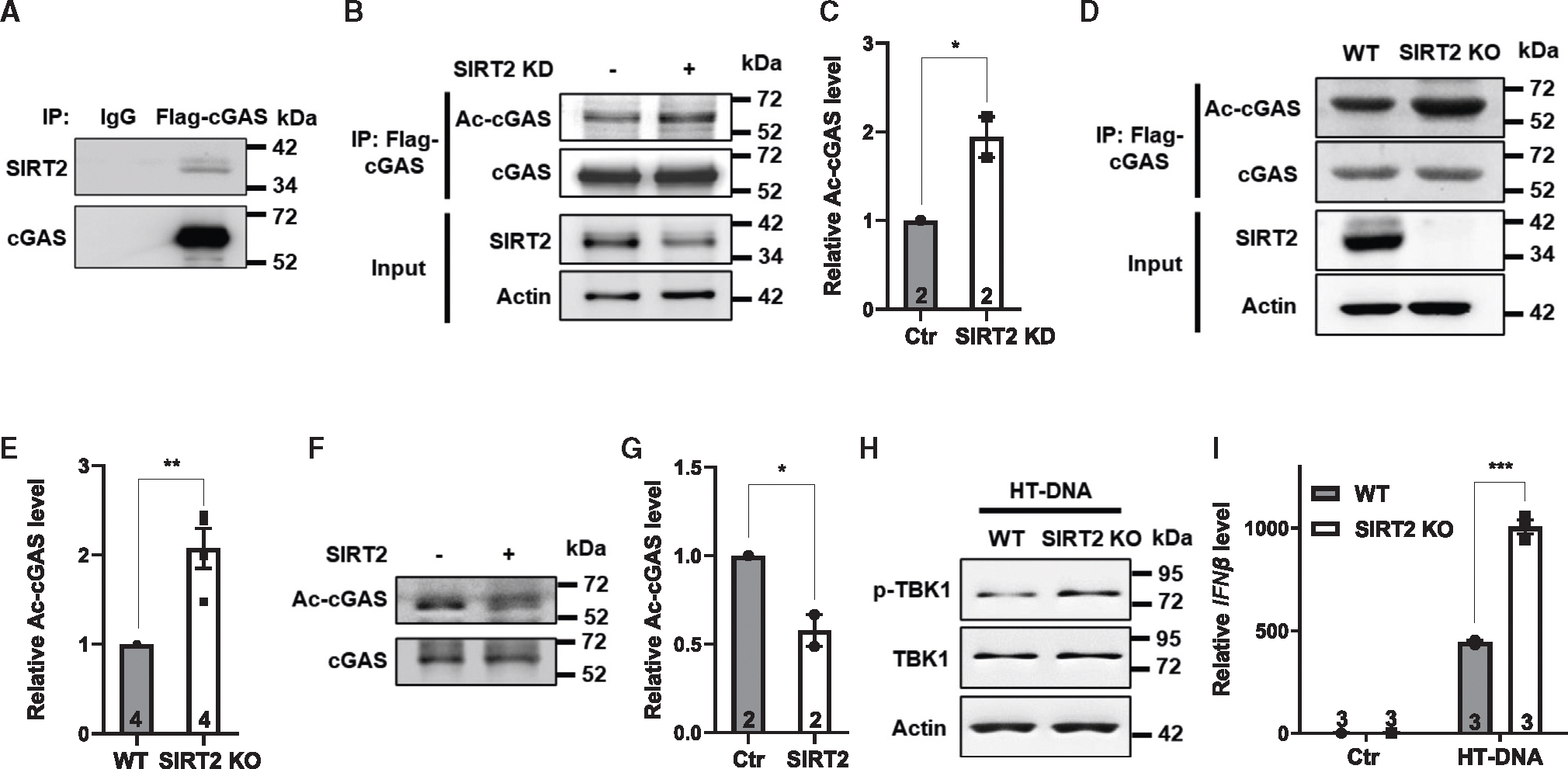
SIRT2 deacetylates cGAS to suppress cGAS activity (A) FLAG-tagged cGAS was transfected into HEK293T cells, treated with HT DNA, and immunopurified, followed by western blot analysis of SIRT2. (B and C) FLAG-tagged cGAS was immunopurified from SIRT2 knockdown and control HEK293T cells, followed by western blot analysis of lysine acetylation (B) and quantification (C). (D and E) WT and SIRT2 KO immortalized myeloid progenitor cells were transduced with FLAG-tagged cGAS retrovirus and treated with HT DNA. FLAG-tagged cGAS was immunopurified, followed by Western blot analyses (D). Quantification of 4 experiments is shown in (E). (F and G) In an *in vitro* deacetylation assay, immunopurified cGAS was incubated with or without immunopurified SIRT2, followed by western blot analysis of lysine acetylation (F) and quantification (G). (H and I) BMDMs derived from WT or SIRT2 KO mice were treated with or without HT DNA. Shown are western blot analyses of TBK1 and phosphorylated TBK1. Actin was used as a control (H). Quantitative real-time PCR analyses for the mRNA levels of IFNβ are shown in (I). Data are mean ± SEM. *p < 0.05, **p < 0.01, ***p < 0.001. *n* = 2–4 independent experiments. See also Figures S4 and S5.

We next examined how SIRT2 suppresses cGAS activity. cGAS is regulated by post-translational acetylation in cells.^45–47^ Acetylation of residues within the DNA binding domain blocks the interaction with DNA and suppresses cGAS activation, while acetylation of lysine residues outside the DNA binding domain promotes cGAS activation.^45–47^ First, we probed SIRT2-cGAS interaction in cells. Western blot analyses of the immunoprecipitates from FLAG-tagged cGAS-transfected cells revealed that SIRT2 was physically associated with cGAS in cells (Figure 3A). Next, we transfected FLAG-tagged cGAS into SIRT2 knockdown or control HEK293T cells and immunopurified FLAG-tagged cGAS. Western blot analyses of immunoprecipitates showed increased acetylation levels of cGAS in SIRT2 knockdown cells (Figures 3B and 3C). Increased cGAS acetylation was also detected in SIRT2 KO immortalized myeloid progenitor cells compared to WT controls (Figures 3D and 3E). Furthermore, in an *in vitro* deacetylation assay using immunopurified cGAS and SIRT2, acetylation levels of cGAS were reduced in the presence of SIRT2 (Figures 3F and 3G). Together, these data suggest that SIRT2 interacts with cGAS and targets cGAS for deacetylation.

To further determine how SIRT2 regulates cGAS activity, we stimulated bone marrow-derived macrophages (BMDMs) derived from WT and SIRT2 KO mice with herring testis (HT) DNA. SIRT2 KO BMDMs showed increased phosphorylation of TBK1, a kinase downstream of cGAS signaling^48,49^ (Figure 3H), and the downstream transcription factor IRF3^50^ (Figure S4A) and elevated expression of cytokines (Figures 3I, S4B, and S4C) compared to WT controls. The expression of cGAS was comparable in WT and SIRT2 KO BMDMs (Figure S4D). In SIRT2-overexpressing THP1-derived macrophages (Figure S5A), IRF3 phosphorylation was suppressed in response to HT DNA stimulation (Figure S5B). Conversely, in SIRT2 knockdown THP1-derived macrophages (Figure S5C), IRF3 phosphorylation and IFNβ expression were increased upon stimulation with HT DNA (Figures S5D and S5E) but not with cyclic guanosine monophosphate-adenosine monophosphate (cGAMP), a second messenger produced downstream of cGAS activation (Figure S5F),^50–52^ indicating that the inhibitory effect of SIRT2 on inflammation is mediated through blocking cGAS activity.

### Reduced SIRT2 expression contributes to aging-associated cGAS activation

The expression of SIRT2 was reduced in BMDMs from old (24 months old) mice compared to those from young (5 months old) mice (Figure S6A), suggesting that SIRT2 is suppressed with aging, consistent with previous reports.^17,29^ Because SIRT2 inhibits cGAS activity (Figures 3H, 3I, S4, and S5), we tested whether cGAS signaling is activated during aging by comparing cGAS signaling in BMDMs from young and old mice. Upon stimulation by HT DNA, BMDMs from old mice exhibited a greater increase in cGAS activity when compared to those from young mice, as indicated by phosphorylation of TBK1 (Figure S6B) and the production of type I IFNs and inflammatory cytokines (Figures S6C–S6E). In the absence of HT DNA, cytokine production was comparable between macrophages derived from young and old mice (Figures S6C–S6E). The expression of cGAS was unaltered in young and old macrophages (Figure S6F). These data suggest dysregulated cGAS signaling during aging that increases its sensitivity to cytosolic DNA.

**Figure 4.**
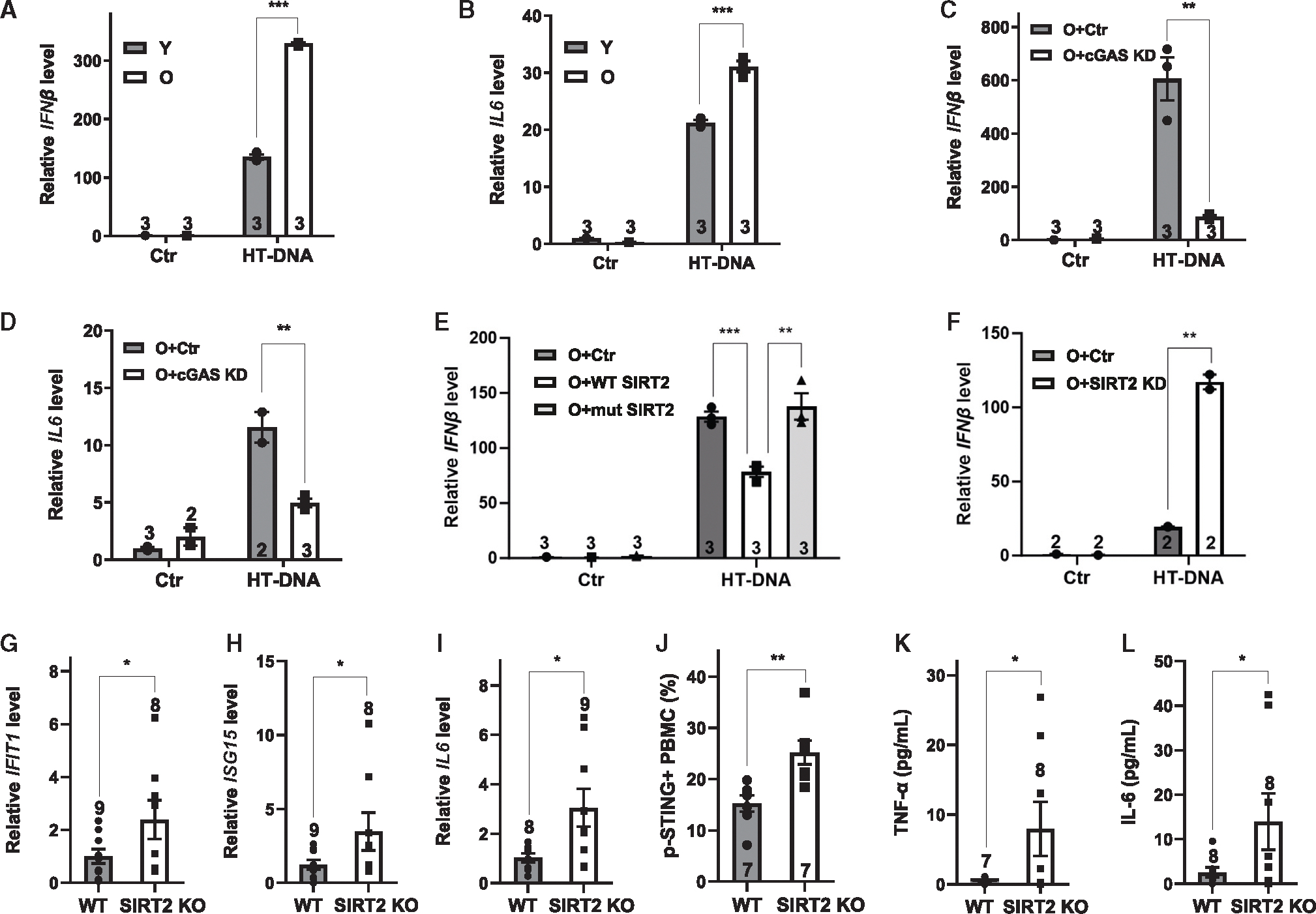
Reduced SIRT2 expression contributes to aging-associated cGAS activation (A and B) Quantitative real-time PCR analyses of the mRNA levels of the indicated genes in macrophages derived from immortalized myeloid progenitors of young (5 months old) or old (27 months old) mice treated with or without HT DNA. *n* = 3 independent experiments. (C and D) Quantitative real-time PCR analyses of the mRNA levels of the indicated genes in macrophages derived from immortalized myeloid progenitors of old mice (27 months old) with or without cGAS shRNA with or without HT DNA treatment. (E) Immortalized myeloid progenitors from old mice (27 months old) were transduced with control or WT or catalytically mutant SIRT2-expressing lentivirus, differentiated into macrophages, and treated with HT DNA or left untreated. The mRNA levels of IFNβ were analyzed by quantitative real-time PCR. (F) Quantitative real-time PCR analyses of the mRNA levels of IFNβ in macrophages derived from immortalized myeloid progenitors of old mice (27 months old) with or without SIRT2 shRNA with or without HT DNA treatment. *n* = 3 independent experiments. (G–I) The mRNA levels of the indicated genes in PBMCs from aged (20 months old) WT or SIRT2 KO mice were analyzed by quantitative real-time PCR. *n* = 9,8 mice/group. (J) Phosphorylation of STING in PBMCs from aged (20 months old) WT or SIRT2 KO mice was analyzed by flow cytometry. *n* = 7 mice/group. (K and L) Levels of inflammatory cytokines in the serum of 12- to 17-month-old WT and SIRT2 KO mice. *n* = 7, 8 mice/group (K) and *n* = 8 mice/group (L). Data are mean ± SEM. *p < 0.05, **p < 0.01, ***p < 0.001. See also Figure S6.

We next confirmed these results using macrophages derived from immortalized myeloid progenitors of young and old mice. These cells, which model primary macrophages isolated from young and old mice, have been used to study aging-associated changes in macrophages^17^ and are amenable to genetic manipulation, unlike primary macrophages. Macrophages derived from immortalized myeloid progenitors of old mice showed increased production of cytokines compared to those from young mice upon stimulation with HT DNA (Figures 4A and 4B), which was suppressed by cGAS inactivation (Figures 4C and 4D). To determine whether suppressed SIRT2 expression with aging contributed to aberrantly activated cGAS signaling, we infected immortalized myeloid progenitors from old mice with lentiviruses expressing WT or catalytically mutant SIRT2 or with control lentivirus, induced them to differentiate into macrophages, and subjected them to HT DNA stimulation. While macrophages derived from immortalized myeloid progenitors from old mice showed increased production of cytokines compared to those from young mice (Figures 4A and 4B), overexpression of WT but not mutant SIRT2 reduced this cytokine expression (Figure 4E). Conversely, knocking down the expression of SIRT2 via shRNA resulted in increased cytokine expression (Figure 4F).

To further confirm the relationship between SIRT2 function and aging-associated cGAS activation, we examined the effect of SIRT2 deficiency on the inflammatory response *in vivo*. Peripheral blood mononuclear cells from 2-year-old SIRT2 KO mice showed increased expression of IFN-stimulated genes (Figures 4G–4I) and increased phosphorylation of STING (Figure 4J) compared to control cells from age-matched WT littermates. Inflammatory cytokine protein levels were increased in aged SIRT2 KO mice compared to WT controls (Figures 4K and 4L). Together, these data suggest that reduced SIRT2 expression contributes to aging-associated cGAS activation.

### SARS-CoV-2 ORF3a is sufficient to induce mtDNA release and trigger an inflammatory response via cGAS

The results described thus far show the protective effects of SIRT2 in SARS-CoV-2-infected aged mice and in protection against excessive cGAS-mediated inflammation. We next investigated the basis of cGAS activation in SARS-CoV-2 infection. SARS-CoV-2 infection leads to mitochondrial damage in infected cells in COVID-19 patients, and it is known that the release of endogenous mtDNA provokes activation of the cGAS-STING pathway and type I IFN immunopathology.^30^ While many factors could be critical in the induction of cGAS in infected mice, we next assessed the role of some of the viral factors that have been implicated in the inflammatory response, lung damage, and mortality (ORF3a, E, or ORF8).^53–55^ In particular, deletion of ORF3a in SARS-CoV-2 reduces morbidity and mortality in infected mice.^56^ To determine whether any of these viral proteins are involved in activation of the cGAS-STING pathway, we infected THP1 cells with a control lentivirus and lentiviruses expressing ORF3a, E, or ORF8, followed by differentiation into macrophages. Expression of ORF3a, but not E or ORF8, increased expression of IFNβ (Figure S7A). We therefore focused on the cellular effects of ORF3a.

**Figure 5.**
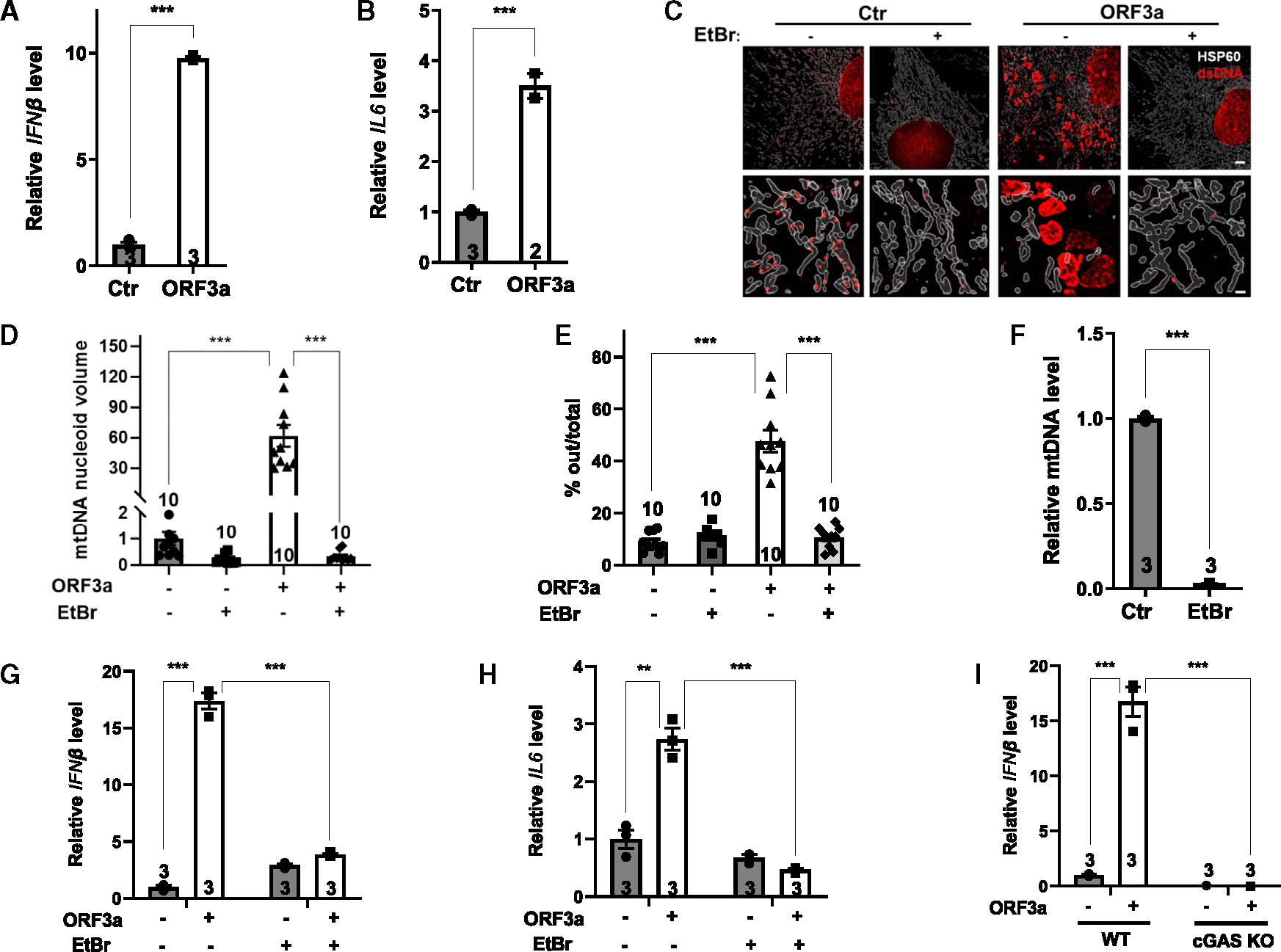
ORF3a induces mtDNA release and triggers the inflammatory response via cGAS MEFs treated with ethidium bromide (EtBr) or left untreated were infected with control or ORF3a-expressing lentivirus. (A and B) Quantitative real-time PCR analyses of the mRNA levels of the indicated genes. (C–E) Lattice SIM 3D super-resolution images for immunostaining with anti-DNA (DNA) and anti-HSP60 (mitochondria) antibodies (C) and quantification of mtDNA nucleoid volume (D) and cytosolic DNA foci outside the mitochondria (E) (scale bars: 5 μm [top] and 0.7 μm [bottom]). *n* = 10 cells/group. (F) Quantitative real-time PCR analyses of mtDNA levels in MEFs treated with EtBr or left untreated. (G and H) Quantitative real-time PCR analyses of mRNA levels of the indicated genes. (I) Quantitative real-time PCR analyses of mRNA levels of IFNβ in WT or cGAS KO MEFs infected with control or ORF3a-expressing lentivirus. Data are mean ± SEM. **p < 0.01, ***p < 0.001. *n* = 2–3 independent experiments. See also Figures S7–S9.

In addition to the induction of IFNs and proinflammatory cytokines (Figures 5A and 5B), ORF3a expression resulted in the appearance of cytosolic DNA outside the mitochondria in control cells but not in cells treated with ethidium bromide, which depletes mtDNA. This observation suggests that the cytosolic DNA is of mitochondrial origin and that ORF3a expression leads to mtDNA stress with enlarged nucleoids and release of mtDNA from the mitochondria (Figures 5C–5E). The effect of ORF3a on mtDNA release and induction of cytokines was observed in multiple cell types tested (Figures 5A–5E, S7A–S7D, S8A, and S9A–S9C). In ethidium bromide-treated cells, mtDNA was depleted (Figures 5F and S9D), and ORF3a expression no longer induced the production of cytokines (Figures 5G, 5H, S9E, and S9F), indicating that ORF3a induces the production of cytokines due to mtDNA release. Induction of IFNβ by ORF3a expression was blunted in cGAS KO mouse embryonic fibroblasts (MEFs) (Figure 5I). Together, these data suggest that ORF3a expression is sufficient to induce mtDNA release, leading to cGAS activation and the production of inflammatory cytokines. In addition to mtDNA release, ORF3a expression also resulted in reduced cellular ATP levels (Figure S8B), reduced mitochondrial membrane potential as assessed by tetramethylrhodamine methyl ester perchlorate expression (Figure S9G), and cell death (Figures S8C and S9H).

### ORF3a is necessary for SARS-CoV-2 to induce mtDNA release and the inflammatory response

**Figure 6.**
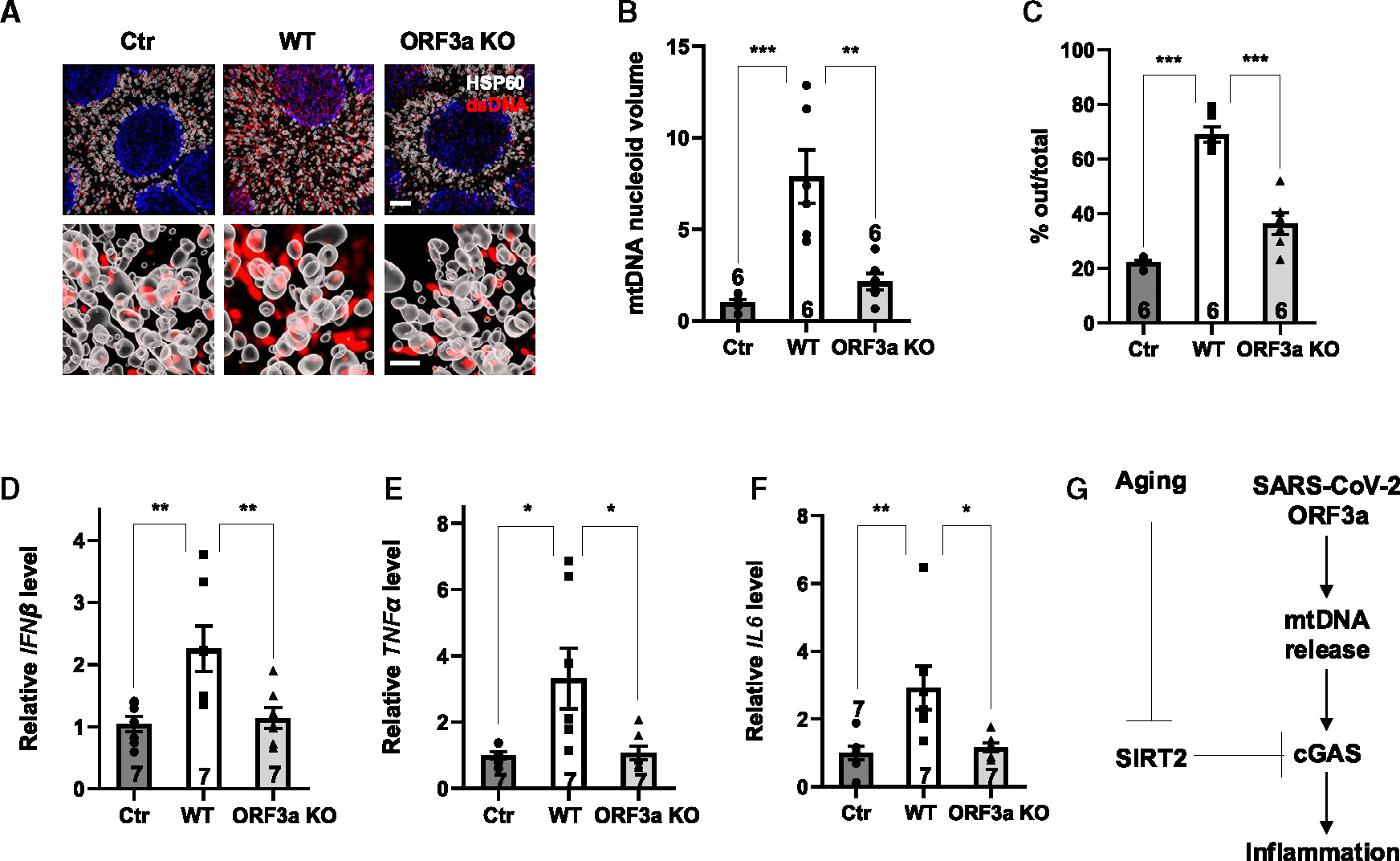
ORF3a is necessary for SARS-CoV-2 to induce mtDNA release and the inflammatory response Primary human airway epithelial cells were infected with 0.1 MOI WT or ORF3a KO SARS-CoV-2 or left uninfected. Cells were analyzed 48 h post infection. (A–C) Lattice SIM 3D super-resolution images for immunostaining with anti-DNA (DNA) and anti-HSP60 (mitochondria) antibodies (A) and quantification of mtDNA nucleoid volume (B) and cytosolic DNA foci outside the mitochondria (C) (scale bar: 2 μm [top] and 0.5 μm [bottom]). *n* = 6 cells/group. (D–F) Quantitative real-time PCR analyses of mRNA levels of the indicated genes. (G) A proposed model. SARS-CoV-2 ORF3a induces mtDNA release, cGAS activation, and inflammation. SIRT2 deactylates cGAS and suppresses cGAS activity. During aging, SIRT2 is suppressed, resulting in aging-associated cGAS activation, inflammation, and increased vulnerability to SARS-CoV-2 infection. Data are mean ± SEM. *p < 0.05, **p < 0.01, ***p < 0.001. *n* = 2 independent experiments.

To validate these results in the context of SARS-CoV-2 infection, we engineered virus deleted in ORF3a expression.^56^ WT and ORF3a KO SARS-CoV-2 have similar replication kinetics.^56^ WT SARS-CoV-2 induced mtDNA stress with enlarged nucleoids, mtDNA release from the mitochondria, and production of inflammatory cytokines (Figures 6A–6F). However, infection with ORF3a KO SARS-CoV-2 resulted in greatly reduced, if any, mtDNA stress, mtDNA release from the mitochondria, and inflammatory response (Figures 6A–6F). Thus, ORF3a is not only sufficient but also necessary for SARS-CoV-2 to induce mtDNA release and the inflammatory response.

### ORF3a is sufficient to cause lung inflammation and immunopathology

**Figure 7.**
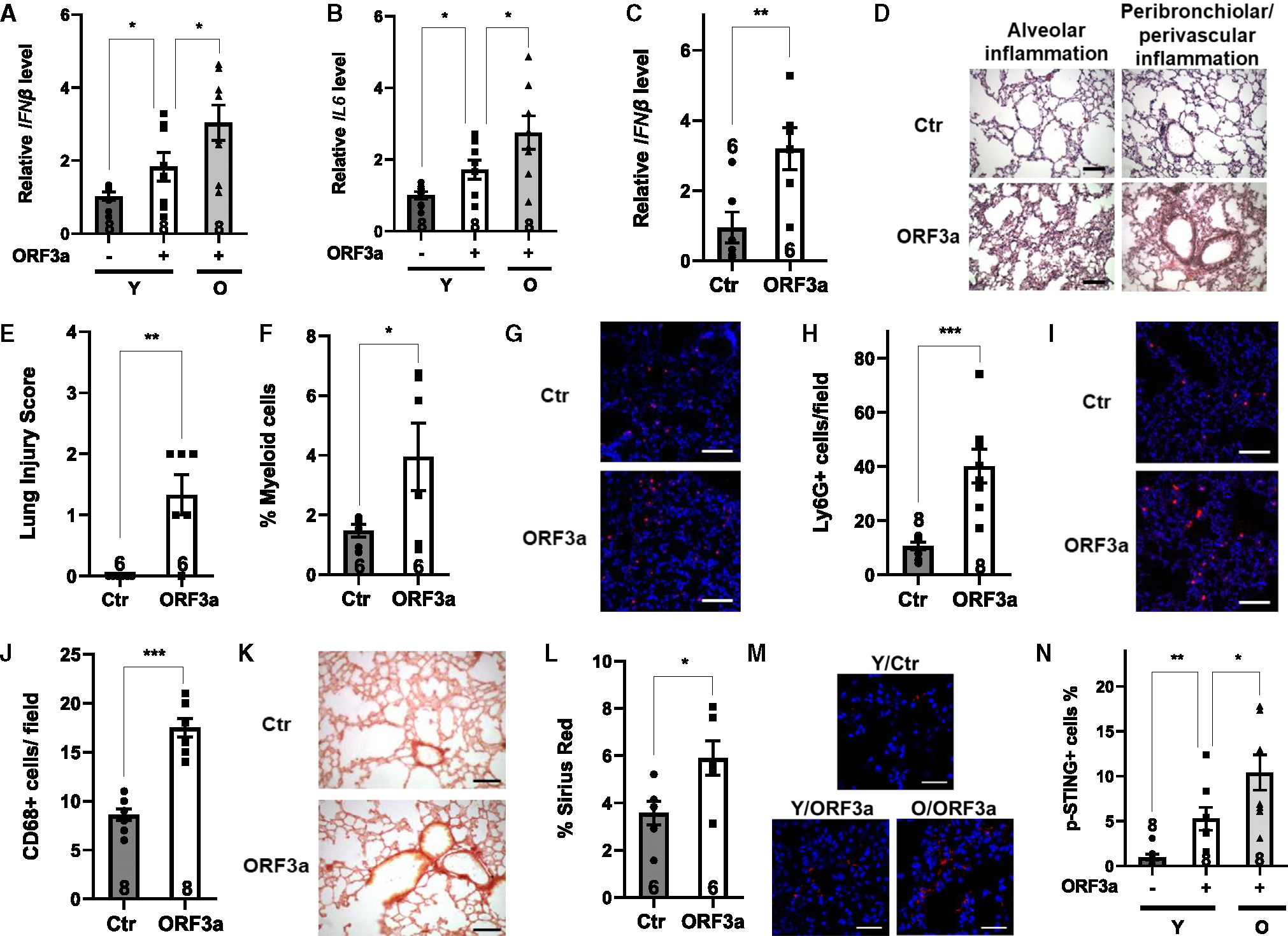
ORF3a triggers lung inflammation and immunopathology Young (5 months old) or old (24 months old) mice were inoculated intranasally with control or ORF3a-expressing AAV6. Mice were analyzed 3 weeks later. *n* =2 independent experiments. (A and B) Quantitative real-time PCR analyses of the mRNA levels of the indicated genes in BALF cells of young and old mice. *n* = 8. (C) Quantitative real-time PCR analyses of the mRNA levels of IFNβ in the lungs of young mice. *n* = 6. (D and E) H&E staining (D) and quantification (E) of lung sections of young mice. Scale bar: 100 μm *n* = 6 mice/group, 10–15 images examined from 3 slides/mouse. (F) Flow cytometry analysis of myeloid cells (Gr1^+^CD11b^+^) in BALF of young mice. *n* = 6. (G and H) Immunostaining for Ly6G^+^ cells (G) and quantification (H) of lung sections. Scale bar: 100 μm *n* = 8 mice/group, 6 images examined from 3 slides/mouse. (I and J) Immunostaining for CD68^+^ cells (I) and quantification (J) of lung sections. Scale bar: 100 μm *n* = 8 mice/group, 4 images examined from 3 slides/mouse. (K and L) Sirius red staining (K) and quantification (L) of lung sections. Scale bar: 200 μm *n* = 6 mice/group, 10 images examined from 3 slides/mouse. (M and N) Immunostaining for phosphorylated STING (M) and quantification (N) of lung sections of young and old mice. Scale bar: 30 μm *n* = 8 mice/group, 5 images examined from 3 slides/mouse. Data are mean ± SEM. *p < 0.05, **p < 0.01, ***p < 0.001. See also Figures S10–S13.

We next determined whether ORF3a alone was sufficient to cause lung inflammation and immunopathology. We expressed ORF3a in the lungs of young WT mice via AAV6-mediated intranasal gene delivery. ORF3a expression led to increased expression of cytokines in the bronchoalveolar lavage fluid (BALF) (Figures 7A and 7B) and in the lungs (Figure 7C). H&E staining of lung sections revealed lung injury in mice expressing ORF3a (Figures 7D and 7E). Flow cytometry analysis showed an increase in myeloid cells (Gr1^+^Mac1^+^) in the BALF of mice expressing ORF3a (Figure 7F). Immunohistochemical staining of lung sections showed a significantly increased number of neutrophils (Ly6G^+^) and macrophages (CD68^+^) upon ORF3a expression (Figures 7G–7J). Pulmonary fibrosis is observed in COVID-19 patients.^57,58^ Increased Sirius red staining, consistent with lung fibrosis, was observed in mice expressing ORF3a (Figures 7K and 7L).

Phosphorylation of STING was increased in the lungs of young mice expressing ORF3a (Figures 7M and 7N), consistent with activation of cGAS signaling by ORF3a. cGAS KO mice were protected from ORF3a-induced lung inflammation and immunopathology, as evidenced by decreased expression of inflammatory cytokines (Figures S10A and S10B), reduced lung injury (Figures S10C and S10D), a decrease in leukocyte infiltration (Figures S10E–S10H), and reduced Sirius red staining (Figures S10I and S10J). These data indicate that ORF3a expression is sufficient to activate cGAS signaling *in vivo* and cause lung inflammation and immunopathology.

Because cGAS activity is increased with aging (Figure S6), we tested whether aged mice were more susceptible to ORF3a-induced lung immunopathology. Upon intranasal inoculation with ORF3a-expressing AAV6, aged mice (24 months old) developed more severe lung inflammation and immunopathology compared to young mice (5 months old), as indicated by the expression of inflammatory cytokines and phosphorylation of STING (Figures 7A, 7B, 7M, and 7N), histological lung injury (Figures S11A and S11B), leukocyte infiltration (Figures S11C–S11F), and lung Sirius red staining (Figures S11G and S11H).

Suppression of cGAS activity by SIRT2 suggested that SIRT2 would suppress ORF3a-induced inflammation and immunopathology. Upon ORF3a expression, cytokines were expressed to a higher level in SIRT2 KO MEFs than in WT MEFs (Figures S12A–S12C). Similarly, SIRT2 knockdown in HeLa cells resulted in expression of higher levels of cytokines than in control cells in response to ORF3a expression (Figures S12D–S12F). ORF3a expression in the lungs via AAV6-mediated gene delivery resulted in a marked increase in inflammation and immunopathology, including increased expression of inflammatory cytokines (Figure S13A), an increase in lung injury (Figures S13B and S13C), leukocyte infiltration (Figures S13D–S13G), and Sirius red staining (Figures S13H and S13I) in SIRT2 KO mice compared to WT mice. Collectively, these results indicate that ORF3a has a role in inducing cGAS activity in SARS-CoV-2-infected cells, with more severe consequences in aged mice.

## DISCUSSION

We identify a molecular mechanism underlying aging-associated inflammation and disease severity in SARS-CoV-2 infected aged mice. We show that SIRT2 activation protects SARS-CoV-2-infected aged mice and that such interventions might be useful in aged SARS-CoV-2-infected patients. A significant portion of aged SIRT2 KO mice died upon infection with a sublethal dose of SARS-CoV-2. Conversely, delivery of an NAD^+^ booster significantly improved the survival of aged mice after a lethal infection. 78c has been used in previous aging studies to boost NAD^+^ levels, increase sirtuin activity, and extend lifespan and healthspan.^44,59–61^ Of note, SIRT2 expression is significantly lower in COVID-19 patients.^62,63^ In one study, lung specimens derived from autopsies of 59- to 77-year-old patients who died of severe COVID-19 showed reduced levels of SIRT2 compared to samples from age-matched control individuals.^62^ SIRT2 levels in the plasma of COVID-19 long haulers were also found to be lower compared to healthy subjects.^63^ Several NAD^+^ boosting strategies, such as nicotinamide mononucleotide and nicotinamide riboside, are being used in clinical trials of individuals with COVID-19 (https://clinicaltrials.gov). While many trials are ongoing, completed studies using a mixture containing NAD booster showed efficacy.^64,65^ In one study of patients with a confirmed positive PCR test for COVID-19, the treatment group showed a significantly shorter time to complete recovery, and in another study of patients with persistent moderate/severe fatigue after COVID-19, treatment improved fatigue symptoms and quality of life.

Our results suggest that the sensitivity of cGAS to cytosolic DNA, such as that observed in SARS-CoV-2-infected cells, is enhanced with aging due to inactivation of SIRT2, contributing to aging-associated inflammation. Our finding complements recent studies demonstrating that cGAS signaling is linked to cellular senescence- and aging-associated inflammation due to the accumulation of cytosolic DNA^66–70^ and highlights a critical role of cGAS signaling in aging-associated inflammation. In addition to suppressing aberrant activation of cGAS signaling during aging, SIRT2 also suppresses aging-associated activation of the NLRP3 inflammasome^17^ and nuclear factor κB.^71^ Both of these pathways are activated in SARS-CoV-2-infected cells and contribute to pathogenesis.^72,73^ Activation of inflammasomes is critical for cell death in SARS-CoV-2-infected lung cells.^73^ Inflammasome activation also occurs after abortive infection of myeloid cells as part of PANoptosis (cell death with features of apoptosis, necroptosis, and pyroptosis) and pro-inflammatory cytokine induction.^72^ Thus, SIRT2 represents a nodal control point for aging-associated inflammation.

Diminished expression of SIRT2 is not the only factor involved in age-dependent increased disease severity in SARS-CoV-2 infected mice. We have shown previously that age-dependent increases in expression of prostaglandin D2 (PGD_2_) and a phospholipase A2 that is upstream in the arachidonic pathway, phospholipaseA2 group 2D, contributes to suboptimal immune responses in aged but not young mice.^27^ Targeting PGD_2_ binding to its receptor on myeloid cells enhanced survival of SARS-CoV-2-infected mice. Another therapeutic strategy might be to target both of these distinct pathways that contribute to severe disease in aged mice.

ORF3a encodes the largest accessory protein in the SARS-CoV-2 genome. Deletion of ORF3a in both SARS-CoV and SARS-CoV-2 reduces morbidity and mortality,^53,56^ suggesting a key role of ORF3a in conferring virulence. ORF3a has been reported to serve as an ion channel,^74^ although a recent report concluded that ORF3a is not an ion channel but, rather, interacts with proteins associated with trafficking proteins to enhance virus replication.^75^ Another study suggested that ORF3a inhibited STING-mediated autophagy to enhance virus replication.^76^ Our study offers additional mechanistic insight into how ORF3a contributes to pathogenicity. We show that SARS-CoV-2 ORF3a is sufficient to trigger mtDNA release and induce the production of type I IFNs and lung immunopathology in a cGAS- and age-dependent manner, with protection mediated by SIRT2. While additional studies of ORF3a function are required, our findings shed light on the mechanistic basis of aging-associated susceptibility to SARS-CoV-2 infection and have implications for novel therapeutic strategies for efficient treatment of COVID-19, especially in highly vulnerable aged populations.

### Limitations of the study

It will be important to extend the study to different strains of mice and to additional animal species. A limitation is that all experiments were performed with ancestral strains of virus. Other SARS-CoV-2 variants will need to be analyzed in the future. Finally, it will be important to assess the degree to which cGAS inhibition diminishes morbidity and mortality in patients with COVID-19.

## RESOURCE AVAILABILITY

### Lead contact

Requests for further information, resources, and reagents should be directed to and will be fulfilled by the lead contact, Danica Chen (danicac@berkeley.edu).

### Material availability

This study did not generate new unique reagents.

### Data and code availability

- All data associated with the study can be found in the figures and supplemental information.
- This paper does not report original code.
- Any additional information required to reanalyze the data reported in this paper is available from the lead contact upon request.

## STAR★METHODS

### EXPERIMENTAL MODEL AND STUDY PARTICIPANT DETAILS

#### Mice

SIRT2 KO mice have been described previously.^17,29^ cGAS KO mice were purchased from the Jackson Laboratory. For experiments using young and old mice, C57BL/6 mice were obtained from the National Institute on Aging. 78c (MedChemExpress LLC, #HY-123999) was administered to mice by intraperitoneal injection (10 mg/kg/dose) twice daily. Control mice received vehicle (5% DMSO, 15% PEG400, 80% of 15% hydroxypropyl-g-cyclodextrin (in citrate buffer pH 6.0)) injections. Mice were housed on a 12:12 h light:dark cycle at 25°C with *ad libitum* access to water and standard laboratory chow diet provided by LabDiet (0007688). All animal procedures were in accordance with the Animal Care Committee at the University of California, Berkeley, and the University of Iowa Animal Care and Use Committee and met stipulations of the Guide for the Care and Use of Laboratory Animals.

#### SARS2-N501Y_MA30_

SARS2-N501Y_MA30_ was isolated and propagated as described previously.^27^ SARS-CoV-2 ORF3 KO was generated and propagated as previously described.^56^

#### Mouse infection with SARS2-N501Y_MA30_ and Tissue Collection

Mice were anaesthetized with ketamine–xylazine and infected intranasally with the indicated amount of virus in a total volume of 50 μL DMEM. Animal weight and health were monitored daily. All experiments with SARS2-N501Y_MA30_ were performed in a biosafety level 3 (BSL3) laboratory at the University of Iowa. At the indicated times, mice were euthanized and perfused transcardially with PBS. Organs were collected and homogenized before clarification by centrifugation.

#### Viral titration

Supernatants of homogenized tissues were serially diluted in DMEM and inoculated onto VeroE6 cells for 1 h. After removing the inocula, plates were overlaid with 0.6% agarose containing 1xDMEM. After 3 days, plaques were visualized by staining with 0.1% crystal violet. Viral titers were quantified as PFUs per mL tissue.

#### Differentiated primary airway culture

Primary epithelial cells were isolated from the human trachea and bronchi and were grown at an air-liquid interface (ALI) as previously described.^78^ Passage-0 primary differentiated human airway epithelia (HAE) from 7 healthy human donors were obtained from the University of Iowa Cells and Tissue Core. Cell cultures from all 7 donors were infected apically with 0.1 MOI WT or ORF3a-KO SARS-CoV-2 for 48 h at 37°C with 5% CO_2_. At 48h, apical washes and basolateral medium were collected and combined for cytokine measurement. Cells were collected into Trizol or fixed with 2% formaldehyde for RT-qPCR or immunostaining, respectively.

#### Cell culture

MEFs were isolated from embryos at day 12.5–14.5, maintained in DMEM (Gibco) supplemented with 10% FBS (Gibco), and cultured no more than six passages before experiments. BMDMs were differentiated from bone marrow cells using M-CSF (20 ng/mL; PeproTech) for 7 days. Cells were grown in DMEM with 10% FBS and penicillin/streptomycin (Gibco).

THP1 cells, 293T cells, and HeLa cells were acquired from cell culture facility at the University of California, Berkeley. 293T and HeLa cells were cultured in DMEM supplemented with 10% FBS. THP-1 cells were cultured in RPMI-1640 medium supplemented with 10% FBS, 4.5 g/L glucose, 10 mM HEPES, 1.0 mM sodium pyruvate and 0.05 mM β-mercaptoethanol. THP-1 cells were treated with 100 ng/mL PMA (Sigma-Aldrich) for 48 h to induce differentiation. THP-1-derived macrophages were washed with RPMI-1640 medium and allowed to grow in PMA-free culture medium for the next 24 h. All cells were maintained in a 5% CO2 incubator at 37°C. Cell proliferation and survival were scored using a Vi-Cell Analyzer (Beckman Coulter).

Immortalization of myeloid progenitors was performed as described.^17^ Briefly, bone marrow was isolated from the femurs of mice before ammonium-chloride-potassium lysis of red blood cells and centrifugation onto a cushion of Ficoll-Paque. Ficoll purified progenitors were infected with ER-Hoxb8 retrovirus and cultured in myeloid progenitor culture medium (RPMI-1640 with 10% FBS, 1% pen-strep-glutamine, 20 ng/mL GM-CSF, 30mM beta mercaptoethanol, and 1mM estrogen). Immortalized myeloid progenitors were selected by moving nonadherent progenitor cells every 3 days to a new culture well for 3 weeks. Differentiation to macrophages was performed by removal of estrogen from the culture medium.

pLVX-EF1alpha-nCoV2019-orf3a-IRES-Puro, pLVX-EF1alpha-nCoV2019-E-IRES-Puro, pLVX-EF1alpha-nCoV2019-orf8-IRES-Puro and pLVX-EF1alpha-GFP-2xStrep-IRES-Puro were a generous gift from the Hurley lab at the University of California, Berkeley. To generate HeLa, MEFs and THP1 cells with transient GFP or ORF3a expression, cells were infected with lentivirus. For lentivirus packaging, 293T cells were co-transfected with packaging vectors (pCMV-dR8.2 dvpr and pCMV-VSV-G) and pLVX-EF1alpha-nCoV2019-orf3a-IRES-Puro or pLVX-EF1alpha-GFP-2xStrep-IRES-Puro. Viral supernatants were harvested 48 h and 72 h after transfection, concentrated by centrifugation, and resuspended with culture medium specific for each cell line in the presence of 10 μg/mL polybrene, and incubated with cells for transduction, as described previously.^79^ Cells were subjected to another cycle of infection on the next day. Transfections were performed with Lipofectamine 2000 (Invitrogen) or PEI (Polysciences), according to the manufacturer’s instructions.

For generation of stable knockdown cell lines, HeLa, THP1, or immortalized myeloid progenitors were transduced with lentivirus, as described above. A SIRT2 knockdown stable cell line was generated using pSicoR-SIRT2 or control shRNA,^17^ and a cGAS knockdown stable cell line was generated using pLKO.1-cGAS (Sigma, TRCN0000178459) or control shRNA. A SIRT2 overexpression stable cell line was generated using retrovirus pBabe-SIRT2 or control constructs.^17^ Retrovirus was generated by transfecting 293T cells with pBabe retroviral constructs as well as VSV-G and gag/pol expression vectors. 48 h after transduction, cells were selected with puromycin (1 μg/mL for 293T, HeLa and THP1 cells, and 4 μg/mL for IMP) (Sigma).

For HT-DNA stimulation, cells were transfected with 1μg/mL of HT-DNA (Sigma) for 3 h.

For cGAMP stimulation, cells were incubated for 30 min at 37°C with cGAMP in permeabilization buffer (50 mM HEPES pH 7, 100 mM KCl, 3 mM MgCl 2, 0.1 mM DTT, 85 mM sucrose, 0.2% BSA, 1 mM ATP and 0.1 mM GTP) with 1 mg/mL digitonin (Sigma). The permeabilization buffer was replaced with RPMI-1640 medium and cells were cultured for 3 h before analysis. For mtDNA depletion, cells were treated with 400 ng/mL ethidium bromide, which was refreshed every 48 h with medium. At day 6, cells were collected and seeded for infection.

### METHOD DETAILS

#### Quantification of ATP

As described previously,^80^ cells in suspension were mixed with an equal volume of CellTiterGlo following the manufacturer’s instructions (Promega). Luminescence was measured using a luminometer (*SpectraMax i3*, Molecular Devices) to obtain relative luciferase units (RLU).

#### Mitochondrial membrane potential (ΔΨM)

Cells were incubated with 250nM tetra methylrhodamine methyl ester perchlorate (TMRM; Thermo Fisher Scientific, T-668) for 30 min at 37°C. Data were acquired on a flow cytometer (BD Fortessa) and analyzed using FlowJo software.

#### Quantitative real-time PCR

Total RNA was isolated from cells and lung homogenates using Trizol reagent (Invitrogen), converted to complementary DNA using the qScript cDNA SuperMix (Quanta Biosciences) and gene expression was determined by real time PCR using Eva qPCR SuperMix kit (BioChain Institute) on an ABI StepOnePlus system. The primer sequences are listed in Table S1. The 2^−ΔΔCt^ method was used to analyze gene expression fold change after normalization with GAPDH and then to the controls. For relative quantification of mtDNA, whole-cell DNA was isolated using DNeasy Blood (Qiagen) and normalized with mTert or hβ-actin and then to the controls.

#### AAV6 packaging

293T cells were transfected with pAAV-CAG-ORF3a and the packaging plasmid pDGM6 (addgene; #110660),^77^ which encodes the AAV serotype 6 capsid. An AAV production method based on chloroform extraction was followed.^81^ AAV vector titers were determined by qPCR as described.^82^ Control virus was produced using pAAV-CAG-GFP (Vigene Biosciences, CV17169-AV6).

#### Intranasal delivery of AAV6V

AAV6 was administered to the respiratory tract of mice by modified intranasal method as described.^83^ Briefly, mice were anesthetized with isoflurane in O2. Using the right hand one individual restrained the mice at a 45°C angle and with the left hand the mouth was gently pinched shut. A second individual delivered a total of 6.5 × 10^11^ vector genomes (vg) in a total volume of 80 μL, delivered dropwise over the nares in 2 anesthesial periods (40 μL each). Drops were passively inhaled.

#### Isolation of mouse peripheral blood mononuclear cells (PBMC) from whole blood

Mouse PBMC were isolated from fresh whole blood collected by submandibular bleeding. Approximately 100 μL of blood per mouse was collected in EDTA tubes. Red blood cells (RBC) were lysed with ACK Lysing Buffer (Gibco) and the resulting cell pellet was used for RNA extraction.

#### Flow cytometry analysis

For analysis of BALF, mice were sacrificed using isoflurane, followed by cannulation of the trachea with a 22-G catheter. BAL was performed with two washes of 0.5 mL sterile PBS. BALF was centrifuged, and single-cell suspensions were treated with ACK lysing buffer (Gibco). The remaining cells were resuspended in PBS. Cells were incubated with antibodies against the following markers: FITC anti-Gr1 (BioLegend, 108406), PE anti-CD11b (BioLegend, 101208). Cells were stained for 15 min at 4°C and washed before resuspension in PBS supplemented with 2% FBS. Flow cytometry data were acquired on a flow cytometer (BD Fortessa) and analyzed using FlowJo software.

For analysis of peripheral blood, blood was subjected to lysis using 1X BD Pharm Lyse Lysing Buffer (BD Biosciences, #555899) for 5 min at room temperature and washed with PBS once. Cells were resuspended in 0.5mL of fixation buffer (BioLegend), and incubated at room temperature for 20 min and washed with 1X perm/wash buffer (BioLegend). Cells were incubated with anti-p-STING antibody (1:300; Cell Signaling, #19781S) at 4°C for 30 min and washed with 1X perm/wash buffer (BioLegend). Cells were then incubated with Alexa Fluor 568-anti Rabbit antibody (1:500; A11036 Invitrogen) at room temperature for 30 min and washed with PBS before flow cytometry analysis.

#### Immunofluorescence microscopy

For microscopy images of THP1, HeLa, MEF and 293T cells, cells were grown on coverslips and transfected, treated, or infected as described above. After washing in PBS, cells were fixed with 4% formaldehyde for 10 min, permeabilized with 0.5% Triton X-100 in PBS for 5 min, and blocked with PBS-0.05% Tween 20 containing 10% FBS for 1 h. Cells were stained with primary antibodies rabbit anti-HSP60 (1:400, CST, 12165S) and mouse anti-dsDNA (1:1000, Abcam, ab27156) overnight at 4°C, followed by secondary antibodies (Alexa Fluors, Invitrogen) for 60 min. Cells were imaged on a Zeiss Elyra 7 inverted system and a 63× objective. The 3D image stacks of the cells were collected using the Zeiss Elyra 7 Lattice SIM system and processed using Zeiss Zen Black software in SIM2 mode. These images were visualized in 3D using IMARIS (v.10.0.1, Bitplane). For cytosolic DNA quantification inside and outside the mitochondria, a surface was created using the HSP60 channel and spots were generated from the dsDNA channel. Huygens Professional (v.23.04, SVI) was used for colocalization analysis of the DNA channel to the mitochondria channel, using the Costes method of thresholding and Object Pearsons method for colocalization. Imaris was used to generate a colocalization channel and surface model from this colocalization result. Then, images were analyzed using IMARIS where a surface was created using the dsDNA channel (total dsDNA cytosolic) and another surface was created corresponding to dsDNA colocalizing with mitochondria (dsDNA inside mitochondria).

OCT-embedded lungs were sectioned at a thickness of 10 μm. Sections were washed with PBS and incubated for 1 h in blocking solution (0.3% H_2_O_2_, 0.2% Triton X-100, 2% goat serum in PBS). The slides were incubated with anti-Ly-6G antibody (1:100, BioLegend, #127605 or 1:250, BioLegend, #127602) or anti-CD68 antibody (1:500, Bio-Rad, MCA1957GA) or anti-p-STING antibody (1:400, Cell Signaling, #19781S) overnight at 4°C. After washes, the sections were incubated with secondary antibodies for 1 h. Lung sections were imaged on a Zeiss LSM 880 FCS inverted confocal microscope or a Zeiss LSM 710 confocal microscope. The positive cells were manually counted or counted using Image J.

#### Histopathologic analysis

The lungs were fixed with 4% formaldehyde at 4°C overnight, transferred to 30% sucrose in PBS, and incubated at 4°C for 2 days for cryoprotection. The lung tissues were frozen in Tissue-Plus O.C.T. Compound (Fisher Scientific) and 10 mm-thick sections were cut using a cryostat. Sections were stained with Hematoxylin and Eosin (H&E) (Abcam, ab245880) and imaged with a Zeiss AxioImager M2 microscope. H&E-stained sections were examined using a semiquantitative, 5-point grading score (0-normal, 1-mild, 2-moderate, 3-marked, 4-severe) and considering four different histopathological parameters: 1) perivascular inflammation, 2) bronchial or bronchiolar epithelial degeneration or necrosis, 3) bronchial or bronchiolar inflammation, and 4) alveolar inflammation.^84,85^

#### Sirius Red staining

Lung sections were stained with Picro-Sirius Red staining (0.1% Fast Red, Sigma-Aldrich, 365548). Images were taken with a Zeiss AxioImager M2 microscope. Assessment of area stained was done by Image J and % Sirius Red staining area was quantified by Sirius red positive area/total lung tissue area.

#### Immunoprecipitations

Immunoprecipitations were performed as previously described.^86^ MSCV-Flag-cGAS construct, a gift from the Vance lab at the University of California, Berkeley, was transfected into a 293T cell line with stable SIRT2 knockdown or control knockdown 293T cells. Proteins were extracted in lysis buffer (50 mM Tris-HCl pH 7.5, 150 mM NaCl, 10% glycerol, 2 mM MgCl_2_, 1 mM DTT, 1% NP40, 1mM PMSF, and protease inhibitor). Protein extracts were subjected to centrifugation at 14,000 rpm for 10 min. Protein lysates were pre-cleared with protein A/G beads (Santa Cruz Biotechnology) for 1 h before immunoprecipitation with Flag-beads (Sigma) overnight. Flag beads containing immunoprecipitates were washed with lysis buffer and eluted with Flag peptide (Sigma).

WT and SIRT2 KO immortalized myeloid progenitor cells were transduced with MSCV-Flag-cGAS retrovirus and stimulated with 2μg/mL of HT-DNA (Sigma) for 3 h, before immunoprecipitation following the same procedure described above.

#### *In vitro* deacetylation assay

SIRT2-FLAG and cGAS-Flag were immunopurified with anti-FLAG M2 agarose, as described above, and were incubated in deacetylation reaction buffer (50 mM Tris-HCl pH 8.8, 5% glycerol, 50 mM NaCl, 4 mM MgCl_2_, 1 mM DTT, 200 nM TSA) in the presence of 50 μm NAD^+^ at 37°C for 1 h. Acetylated cGAS or cGAS were detected by Western blotting.

#### Western Blot

Cells lysates or immunoprecipitated proteins were resolved on SDS-PAGE and transferred to a nitrocellulose membrane (Bio-Rad). The membranes were probed with antibodies for phospho-IRF3 (CST, 4947S), IRF3 (Abcam, 68481), β-actin (Sigma, A2066), SIRT2 (Proteintech, 66410–1-Ig), cGAS (CST, 15102S), Acetyl-K (CST, 9441s), TBK1 (CST, 3504T), phospho-TBK1 (CST, 5483S). Membranes were then washed and incubated for 60 min with 1:2,000 HRP-conjugated secondary antibody (BioLegend). After further washes, the membranes were exposed to enhanced chemiluminescence substrate (PerkinElmer, NEL103001EA), and visualized using ImageQuant LAS 4000 (GE Healthcare).

#### Mouse serum MultiPlex cytokine measurements

Cytokines in mouse sera were measured with the Luminex Mouse cytokine immunoassay for IL-6 and TNF-α (Millipore, MCYTOMAG-70K) on a Bio-Plex MAGPIX Multiplex Reader according to the manufacturer’s instructions.

### QUANTIFICATION AND STATISTICAL ANALYSIS

The number of biological replicates was chosen based on the nature of the experiments and published papers describing similar experiments. No statistical methods were used to predetermine sample sizes. Sample size (n) can be found on the bar graphs representing the biological replicates (the number of mice) or the technical replicates. Mice were randomized to groups and analysis of mice and tissue samples was performed by investigators blinded to the treatment or the genetic background of the animals. Statistical analysis was performed with Student’s *t*-test, unless specified otherwise, and differences in survival were analyzed by log-rank (Mantel-Cox) tests (GraphPad Prism software). Data are presented as means and error bars represent standard errors, unless specified otherwise. In all corresponding figures, * represents *p* < 0.05. ** represents *p* < 0.01. *** represents *p* < 0.001. ns represents *p* > 0.05.

## Supplementary Material

1

## Figures and Tables

**KEY RESOURCES TABLE:** 

REAGENT or RESOURCE	SOURCE	IDENTIFIER
Antibodies		
HSP60 (D6F1) XP^®^ Rabbit monoclonal antibody	Cell Signaling Technology	Cat# 12165; RRID:AB_2636980
Anti-ds DNA [35I9 DNA] Mouse monoclonal antibody	Abcam	Cat# ab27156; RRID:AB_470907
FITC Ly-6G Rat monoclonal antibody	BioLegend	Cat# 127605; RRID:AB_1236488
Ly-6G Rat monoclonal antibody	BioLegend	Cat# 127602; RRID:AB_1089180
CD68 clone FA-11 Rat monoclonal antibody	Bio-Rad	Cat# MCA1957GA; RRID:AB_324217
Phospho-STING (Ser366) (D7C3S) Rabbit monoclonal antibody	Cell Signaling Technology	Cat# 19781; RRID:AB_2737062
Anti-α-Actin-1 (ACTA1) Rabbit polyclonal antibody	Sigma-Aldrich	Cat# A2066; RRID:AB_476693
SIRT2 Mouse monoclonal antibody	Proteintech	Cat# 66410-1-Ig; RRID:AB_2881782
Phospho-IRF-3 (Ser396) (4D4G) Rabbit monoclonal antibody	Cell Signaling Technology	Cat# 4947; RRID:AB_823547
IRF3 antibody [EPR2418Y] Rabbit monoclonal antibody	Abcam	Cat# ab68481; RRID:AB_11155653
cGAS (D1D3G) Rabbit monoclonal antibody	Cell Signaling Technology	Cat# 15102; RRID:AB_2732795
Acetylated-Lysine Antibody	Cell Signaling Technology	Cat# 9441; RRID:AB_331805
TBK1/NAK (D1B4) Rabbit monoclonal antibody	Cell Signaling Technology	Cat# 3504; RRID:AB_2255663
Phospho-TBK1/NAK (Ser172) (D52C2) XP^®^ Rabbit monoclonal antibody	Cell Signaling Technology	Cat# 5483; RRID:AB_10693472
Goat anti-rabbit IgG antibody Alexa Fluor^®^ 488	Thermo Fisher Scientific	Cat# A32731; RRID:AB_2633280
Goat anti-mouse IgG antibody Alexa Fluor^®^ 568	Thermo Fisher Scientific	Cat# A11031; RRID:AB_144696
Goat anti-Rat IgG Alexa Fluor^™^ Plus 594	Thermo Fisher Scientific	Cat# A48264; RRID:AB_2896333
Goat anti-Rabbit IgG Alexa Fluor^™^ 568	Thermo Fisher Scientific	Cat# A11036; RRID:AB_10563566
HRP Donkey anti-rabbit IgG Antibody	BioLegend	Cat# 406401; RRID:AB_2099368
HRP Goat anti-mouse IgG Antibody	BioLegend	Cat# 405306; RRID:AB_315009
FITC anti-mouse Ly-6G/Ly-6C (Gr-1) Antibody	BioLegend	Cat# 108406; RRID:AB_313371
PE anti-mouse/human CD11b Antibody	BioLegend	Cat# 101208; RRID:AB_312791
Bacterial and virus strains		
SARS2-N501YMA30	Wong et al. 2022^27^	
SARS-CoV-2 ORF3 KO	Silvas et al. 2021^56^	
SARS-CoV-2 WT	Silvas et al. 2021^56^	
Chemicals, peptides, and recombinant proteins		
78c (CD38 inhibitor)	MedChemExpress	Cat#: HY-123999 CAS#:1700637-55-3
Dimethyl Sulfoxide (DMSO)	Sigma-Aldrich	Cat# D8418
Polyethylene glycol 400 (PEG400)	Sigma-Aldrich	Cat# PX1286B
Hydroxypropyl-g-cyclodextrin	Santa Cruz biotechnology	Cat# sc-238090A
Dulbecco’s Modification of Eagle’s Medium	Gibco	Cat# 11995065
Fetal Bovine Serum	Invitrogen	Cat#10437-028
Penicillin Streptomycin Solution (100×)	Invitrogen	Cat# 15140122
RPMI 1640 Medium	Gibco	Cat# 11875093
Penicillin-Streptomycin-Glutamine (100X)	Gibco	Cat# 10378016
Puromycin	Sigma-Aldrich	Cat# P9620
Murine GM-CSF	Peprotech	Cat# 315-03
Estradiol	Sigma-Aldrich	Cat# E8875
Ficoll-Paque plus	GE-healthcare Life Science	Cat# 17144002
2-Mercaptoethanol	Gibco	Cat# 21985023
Mouse M-CSF Recombinant Protein	Peprotech	Cat# 315-02
Glucose Solution	Gibco	Cat# A2494001
Sodium Pyruvate	Gibco	Cat# 11360070
HEPES	Gibco	Cat# 15630080
OptiPRO^™^ SFM	Gibco	Cat# 12309019
PEI MAX^®^	Polysciences	Cat# 24765 (1)
Polybrene^®^	Santa Cruz biotechnology	Cat# sc-134220
Phorbol-12-myristate-13-acetate (PMA)	Sigma-Aldrich	Cat# 524400
Lipofectamine 2000	Invitrogen	Cat# 11668019
ACK Lysing Buffer	Gibco	Cat# A1049201
Deoxyribonucleic acid sodium salt from herring testes	Sigma-Aldrich	Cat# D6898
dGTP Solution	Thermo Scientific	Cat# R0161
ATP	InvivoGen	Cat# tlrl-atpl
2′3′-cGAMP	InvivoGen	Cat# tlrl-nacga23
Digitonin	Sigma-Aldrich	Cat# D141
Ethidium bromide solution	Sigma-Aldrich	Cat# E1385
Tetramethylrhodamine, Methyl Ester, Perchlorate (TMRM)	Invitrogen	Cat# T668
TRIzol Reagent	Invitrogen	Cat# 15596026
Ketamine	Dechra	Cat#NDC59399-114-10
Xylazaine	Dechra	Cat#17033-099-05
Agarose (Low Melt Temperature)	Research Products International	Cat#9012-36-6
Crystal violet	Sigma Aldrich	Cat#C0775
Tissue-Plus^™^ O.C.T. Compound	Fisher Scientific	Cat#23-730-571
qScript^™^ cDNA SuperMix	Quanta Biosciences	Cat# 95048
BD Pharm Lyse^™^ Lysing Buffer	BD Biosciences	Cat# 555899
Fixation Buffer	BioLegend	Cat# 420801
Intracellular Staining Permeabilization Wash Buffer	BioLegend	Cat# 421002
Formaldehyde	Thermo Fisher Scientific	Cat# F79
DAPI (4′,6-diamidino-2-phenylindole, dihydrochloride)	Thermo Fisher Scientific	Cat#62247
Sirius red (direct red 80)	Sigma-Aldrich	Cat# 365548
Protein A/G PLUS-Agarose	Santa Cruz biotechnology	Cat# sc-2003
ANTI-FLAG^®^ M2 Affinity Gel	Sigma-Aldrich	Cat# A2220
Halt^™^ Protease Inhibitor Cocktail	Thermo Fisher Scientific	Cat# 78429
Trichostatin A	Sigma-Aldrich	Cat# T1952
Western (blotting) Lightning Plus-ECL substrate	Perkin Elmer	Cat# NEL103E001EA
3X FLAG^®^ Peptide	Sigma-Aldrich	Cat# F4799
Critical commercial assays		
Eva qPCR SuperMix kit	BioChain Institute	Cat# K5052200
MILLIPLEX^®^ Mouse Cytokine/Chemokine Magnetic Bead Panel	Millipore	Cat# MCYTOMAG-70K
CellTiter-Glo^®^ Luminescent Cell Viability Assay	Promega	Cat# G7570
DNeasy Blood & Tissue Kit	QIAGEN	Cat# 69504
H&E Staining Kit (Hematoxylin and Eosin)	Abcam	Cat# ab245880
Experimental models: Cell lines		
HEK293T	UC Berkeley Cell Culture Facility	
HeLa	UC Berkeley Cell Culture Facility	
THP1	UC Berkeley Cell Culture Facility	
HAE	University of Iowa Cells and Tissue Core	
VeroE6	Perlman lab at the University of Iowa, Iowa City	
Experimental models: Organisms/strains		
Mouse: C57BL/6J	National Institute on Aging	
Mouse: SIRT2 KO	Heetal. 2020^17^	
Mouse: cGAS KO	Jackson Laboratory	
Oligonucleotides		
Primers	Table S1	
Recombinant DNA		
pCMV-dR8.2 dvpr	Heetal. 2020^17^	
pCMV-VSV-G	Heetal. 2020^17^	
pMSCV-Hoxb8	Heetal. 2020^17^	
EcoPac	Heetal. 2020^17^	
pLVX-EF1alpha-nCoV2019-orf3a-IRES-Puro	Hurley lab at the University of California, Berkeley	
pLVX-EF1alpha-nCoV2019-E-IRES-Puro	Hurley lab at the University of California, Berkeley	
pLVX-EF1alpha-nCoV2019-orf8-IRES-Puro	Hurley lab at the University of California, Berkeley	
pLVX-EF1alpha-GFP-2xStrep-IRES-Puro	Hurley lab at the University of California, Berkeley	
pSicoR-SIRT2	Heetal. 2020^17^	
pSicoR	Heetal. 2020^17^	
pLKO.1-cGAS	Sigma-Aldrich	TRCN0000178459
pLKO.5	Sigma-Aldrich	TRCN0000251263
MSCV-Flag-cGAS	Vance lab at the University of California, Berkeley	
pBabe-SIRT2	Heetal. 2020^17^	
pBabe	Heetal. 2020^17^	
gag/pol	Heetal. 2020^17^	
pDGM6	Gregorevic et al. 2004^77^	Addgene, Plasmid: #110660
pAAV-CAG-GFP	Vigene Biosciences	Cat# CV17169-AV6
pAAV-CAG-ORF3a	Vigene Biosciences	
Software and algorithms		
ImageJ		https://imagej.nih.gov/ij/
GraphPad Prism	GraphPad	https://www.graphpad.com/
IMARIS v.10.0.1		Bitplane; http://www.bitplane.com/imaris/imaris
Huygens Professional v.23.04	(Scientific Volume Imaging)	https://svi.nl/Huygens-Professional

## Highlights

- SIRT2 and NAD^+^ boosting protect against aging-associated severe COVID-19
- SARS-CoV-2 ORF3a induces mitochondrial DNA release, cGAS activation, and inflammation
- SIRT2 deacetylates cGAS and inhibits aging-associated cGAS activation and inflammation
