# Supplementary material for: SIRT2 suppresses aging-associated cGAS activation and protects aged mice from severe COVID-19
Source: Cell Rep. Author manuscript; Available in PMC 2025 May 13. (PMC12074670; doi:10.1016/j.celrep.2025.115562)
Supplement: 1 [file NIHMS2076720-supplement-1.pdf]

Cell Reports, Volume *44*

Supplemental information

SIRT2 suppresses aging-associated cGAS activation and protects aged mice from severe COVID-19

Marine Barthez, Biyun Xue, Jian Zheng, Yifei Wang, Zehan Song, Wei-Chieh Mu, Chih-ling Wang, Jiayue Guo, Fanghan Yang, Yuze Ma, Xuetong Wei, Chengjin Ye, Nicholas Sims, Luis Martinez-Sobrido, Stanley Perlman, and Danica Chen

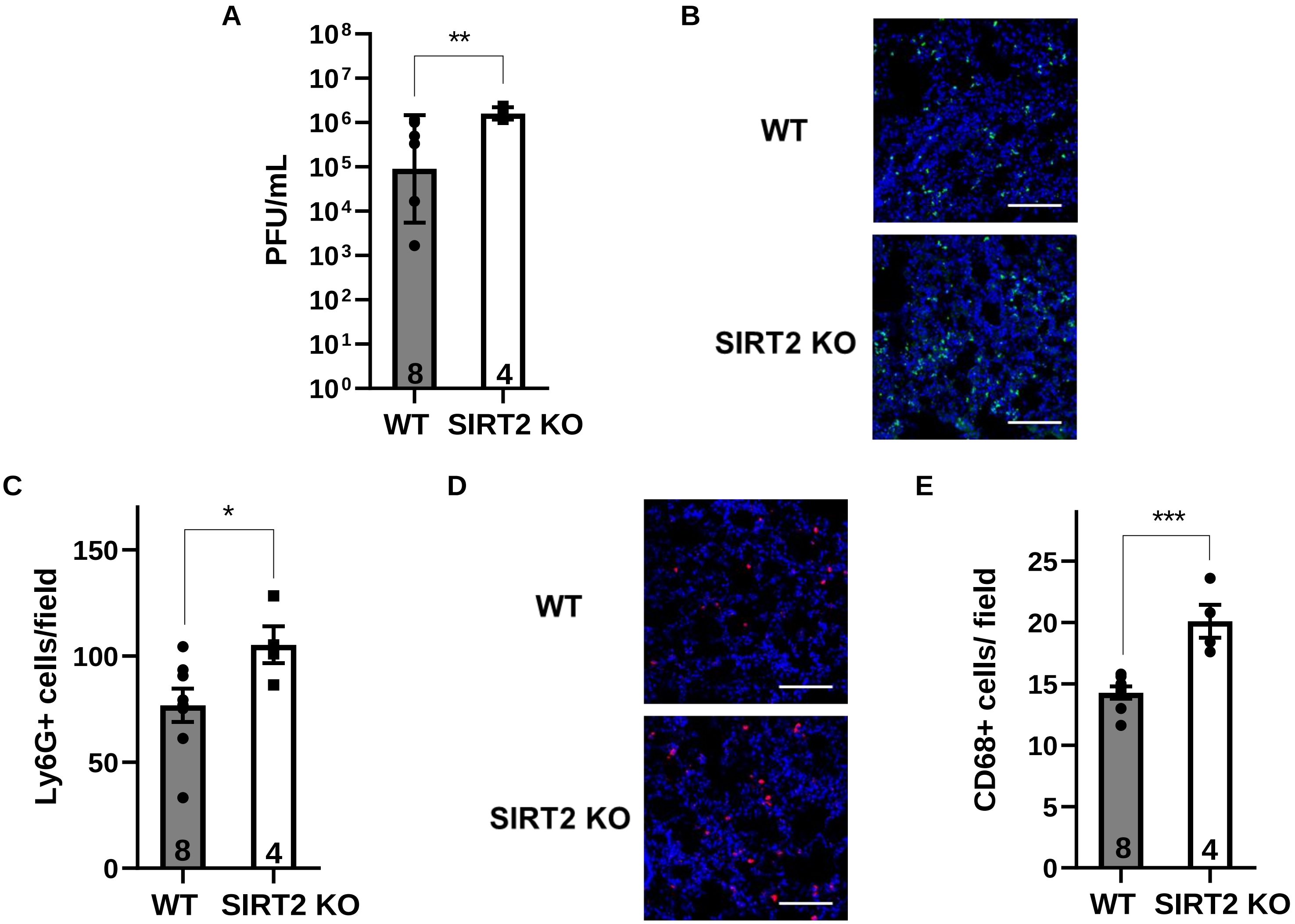

Figure S1. SIRT2 protects aged mice from lethal SARS-CoV-2 infection. Related to Figure 1.

Aged (18-24 months old) WT and SIRT2 KO mice were infected with 1,000 PFUs of SARS2-N501Y$_{MA30}$.

A, Viral titer in lungs 5 dpi. Data are geometric mean ± geometric s.d. Mann-Whitney test.

B, C, Immunostaining for Ly6G+ cells (B) and quantification (C) of lung sections. Scale bar: 100 μm. n=8,4 mice/group, 5 images examined from 3 slides/mouse.

D, E, Immunostaining for CD68+ cells (D) and quantification (E) of lung sections. Scale bar: 100 μm. n=8,4 mice/group, 5 images examined from 3 slides/mouse.

Data are mean ± s.e.m. * $p < 0.05$. ** $p<0.01$. *** $p <0.001$.

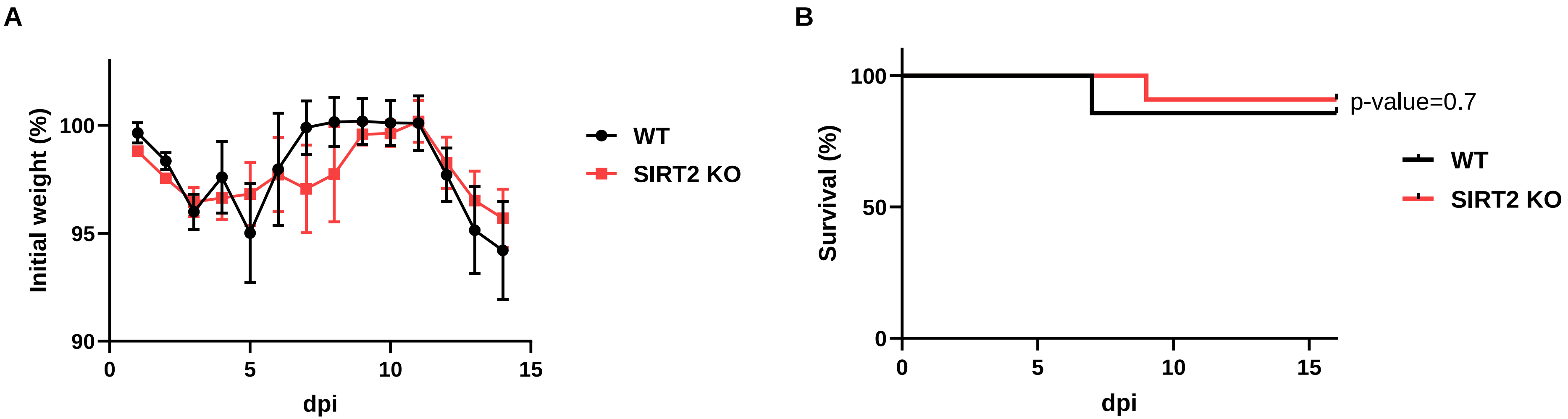

Figure S2. SARS-CoV-2 infection in 10-month-old SIRT2 KO mice. Related to Figure 1.
10-month-old WT and SIRT2 KO mice were infected with 1,000 PFUs of SARS2-N501Y$_{MA30}$.
A, Body Weight. n=7,11 mice/group.
B, Survival curve. n=7,11 mice/group.
Data are mean ± s.e.m.

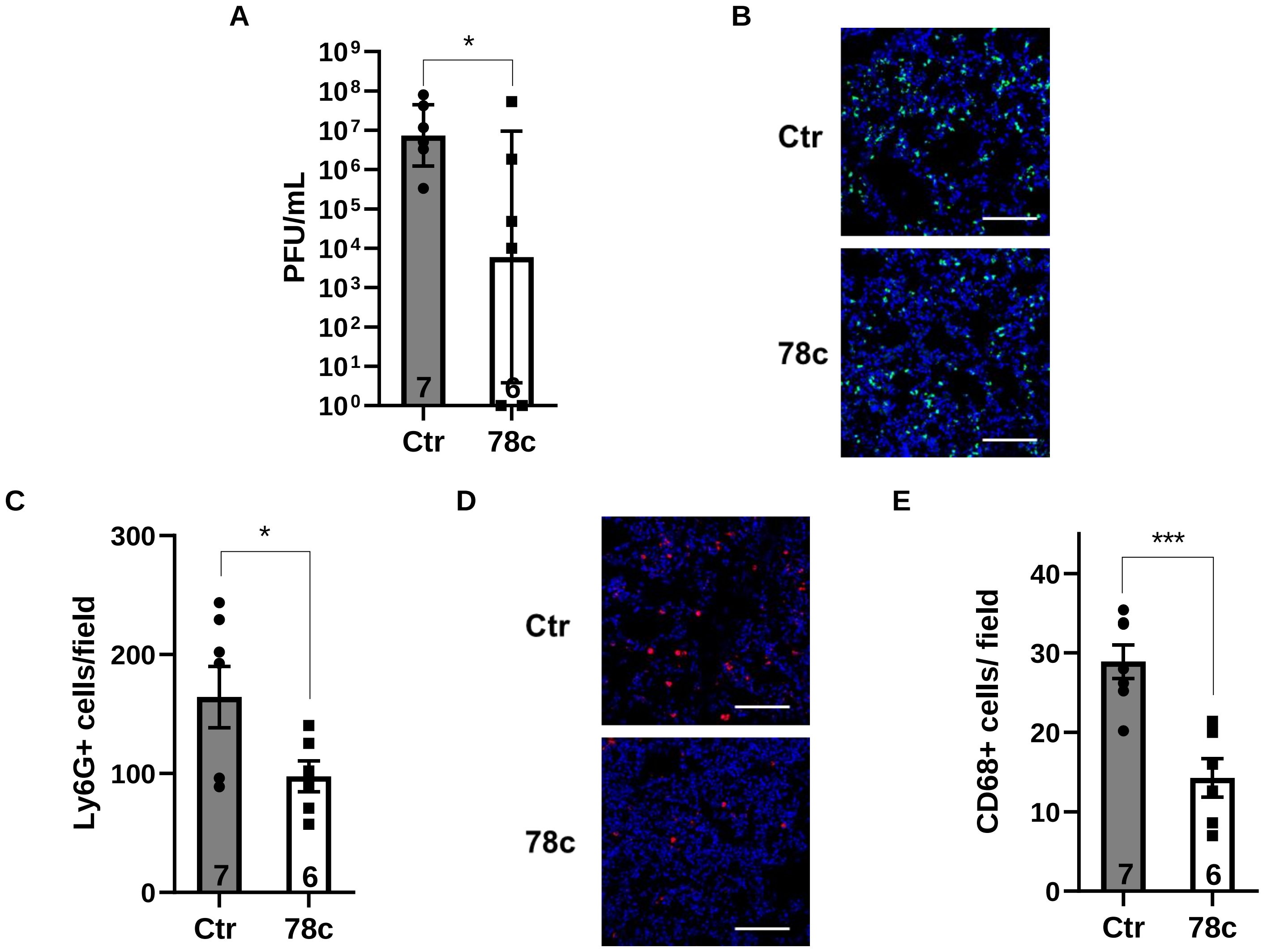

Figure S3. NAD⁺ boosting protects aged mice from lethal SARS-CoV-2 infection. Related to Figure 2.

Aged (24 months old) WT mice infected with 5,000 PFUs of SARS2-N501Y$_{MA30}$ followed by treatment with or without 78c 1dpi.

A, Viral titer in lungs 5 dpi. Data are geometric mean ± geometric s.d. Mann-Whitney test.

B, C, Immunostaining for Ly6G+ cells (B) and quantification (C) of lung sections. Scale bar: 100 μm. n=7,6 mice/group, 5 images examined from 3 slides/mouse.

D, E, Immunostaining for CD68+ cells (D) and quantification (E) of lung sections. Scale bar: 100 μm. n=7,6 mice/group, 5 images examined from 3 slides/mouse.

Data are mean ± s.e.m. * $p < 0.05$. *** $p < 0.001$.

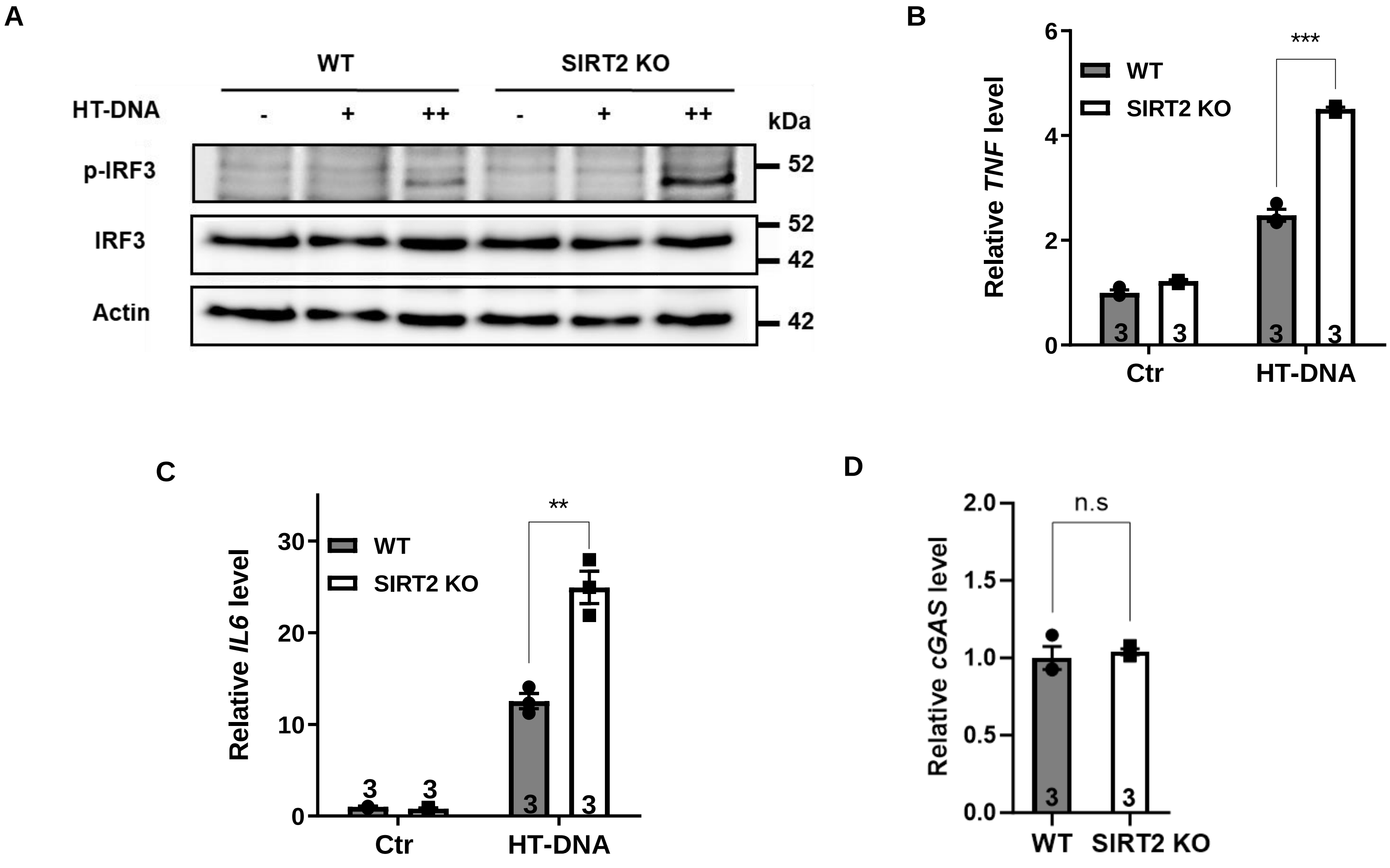

Figure S4. SIRT2 suppresses cGAS activity in BMDMs. Related to Figure 3.

A-D, BMDMs derived from WT or SIRT2 KO mice were treated with or without HT-DNA (+: 0.1 µg/ml; ++: 1 µg/ml).
A, Western blotting analyses of IRF3 and phosphorylated IRF3. Actin was used as a control.
B-D, Quantitative real-time PCR analyses for the mRNA levels of the indicated genes.
Data are mean ± s.e.m. ** $p < 0.01$. *** $p < 0.001$. ns $p > 0.05$. n=2 independent experiments.

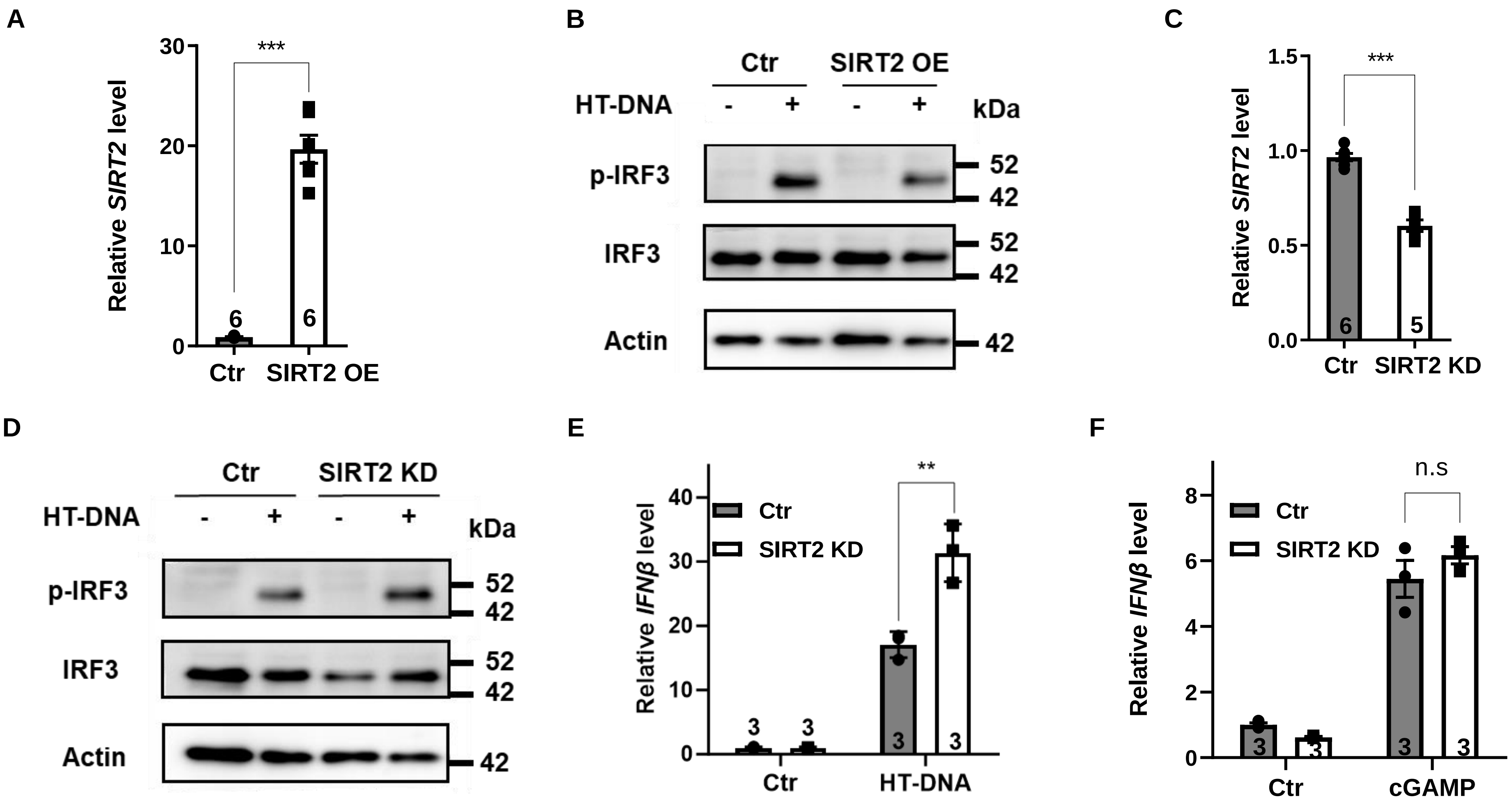

Figure S5. SIRT2 suppresses cGAS activity in THP1 cells. Related to Figure 3.

A, B, Comparison of control and SIRT2 overexpressing THP1-derived macrophages.

A, Quantitative real-time PCR analyses for the mRNA levels of SIRT2.

B, Western blotting analyses of IRF3 and phosphorylated IRF3 upon HT-DNA induction. Actin was used as a control.

C-F, Comparison of control and SIRT2 knockdown THP1-derived macrophages.

C, Quantitative real-time PCR analyses for the mRNA levels of SIRT2.

D, Western blotting analyses of IRF3 and phosphorylated IRF3 upon HT-DNA induction. Actin was used as a control.

E, Quantitative real-time PCR analyses for the mRNA levels of IFNβ upon HT-DNA induction. n=3 independent experiments.

F, Quantitative real-time PCR analyses for the mRNA levels of IFNβ upon cGAMP induction.

Data are mean ± s.e.m. ** $p < 0.01$. *** $p < 0.001$. ns $p > 0.05$.

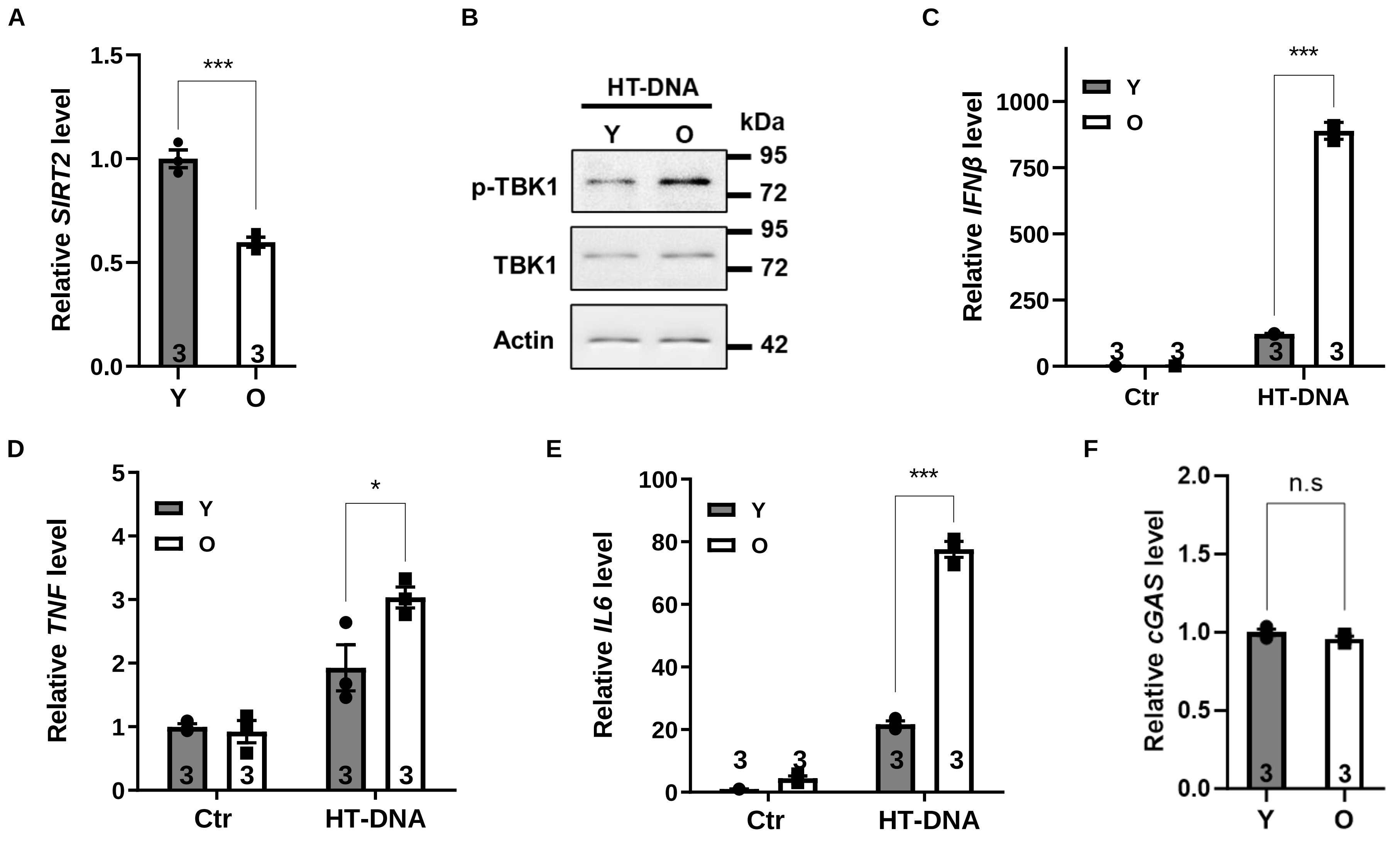

Figure S6. Aberrant activation of cGAS with aging. Related to Figure 4.

Comparison of BMDMs from young (5 months old) or old mice (24 months old) treated with or without HT-DNA. n=2-3 independent experiments.

A, Quantitative real-time PCR analyses for the mRNA levels of SIRT2.

B, Western blotting analyses of TBK1 and phosphorylated TBK1. Actin was used as a control.

C-F, Quantitative real-time PCR analyses for the mRNA levels of the indicated genes.

Data are mean ± s.e.m. * $p < 0.05$. *** $p < 0.001$. ns $p > 0.05$.

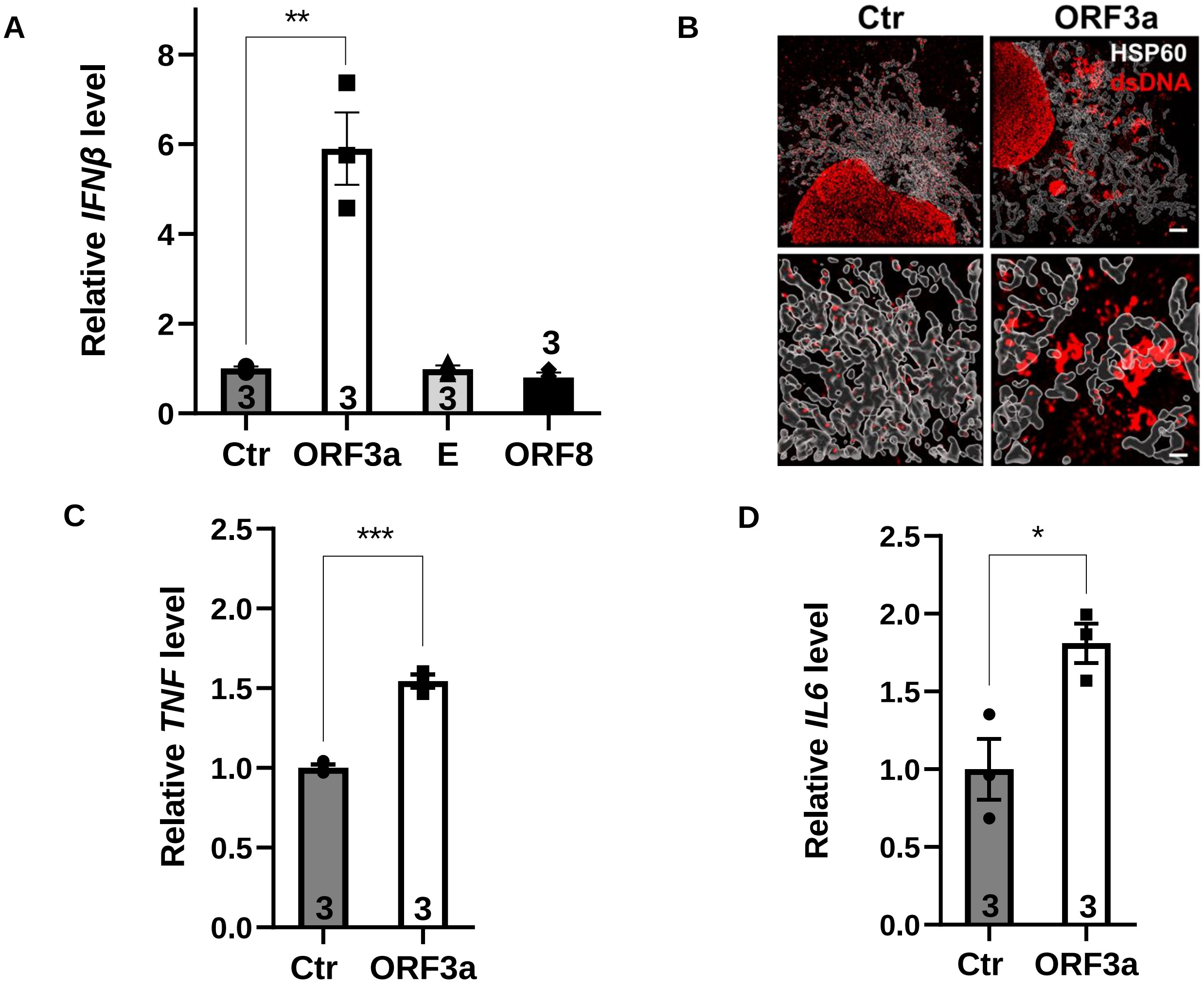

Figure S7. Effects of ORF3a expression in THP-1 cell-derived macrophages. Related to Figure 5.

A, THP1 cells were infected with control lentivirus (Ctr) or lentivirus expressing ORF3a, E, ORF8 followed by differentiation into macrophages. The expression of IFNβ was assessed by quantitative real-time PCR.

B-D, THP1 cells were infected with control lentivirus (Ctr) or lentivirus expressing ORF3a followed by differentiation into macrophages.

B, Lattice SIM 3D super-resolution images for immunostaining with anti-DNA (DNA) and anti-HSP60 (mitochondria) antibodies (Scale bar: 3μm (top) and 0.8μm (bottom)).

C, D, Quantitative real-time PCR analyses for the mRNA levels of the indicated genes.

Data are mean ± s.e.m. * $p < 0.05$. ** $p < 0.01$. *** $p < 0.001$. n=2-3 independent experiments.

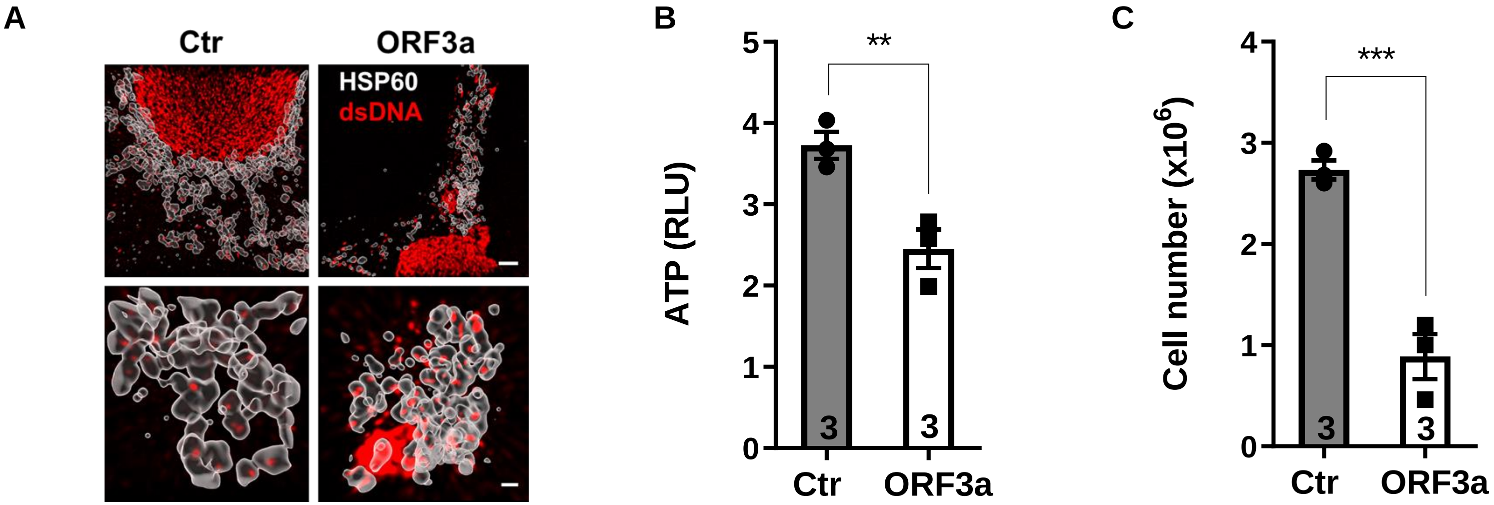

Figure S8. Effects of ORF3a expression in 293T cells. Related to Figure 5.

293T cells were transfected with control or ORF3a-expressing plasmids.

A, Lattice SIM 3D super-resolution images for immunostaining with anti-DNA (DNA) and anti-HSP60 (mitochondria) antibodies (Scale bar: 2µm (top) and 0.4µm (bottom)).

B, Cellular ATP levels.

C, Cell number.

Data are mean ± s.e.m. ** $p < 0.01$. *** $p < 0.001$. n=2-4 independent experiments.

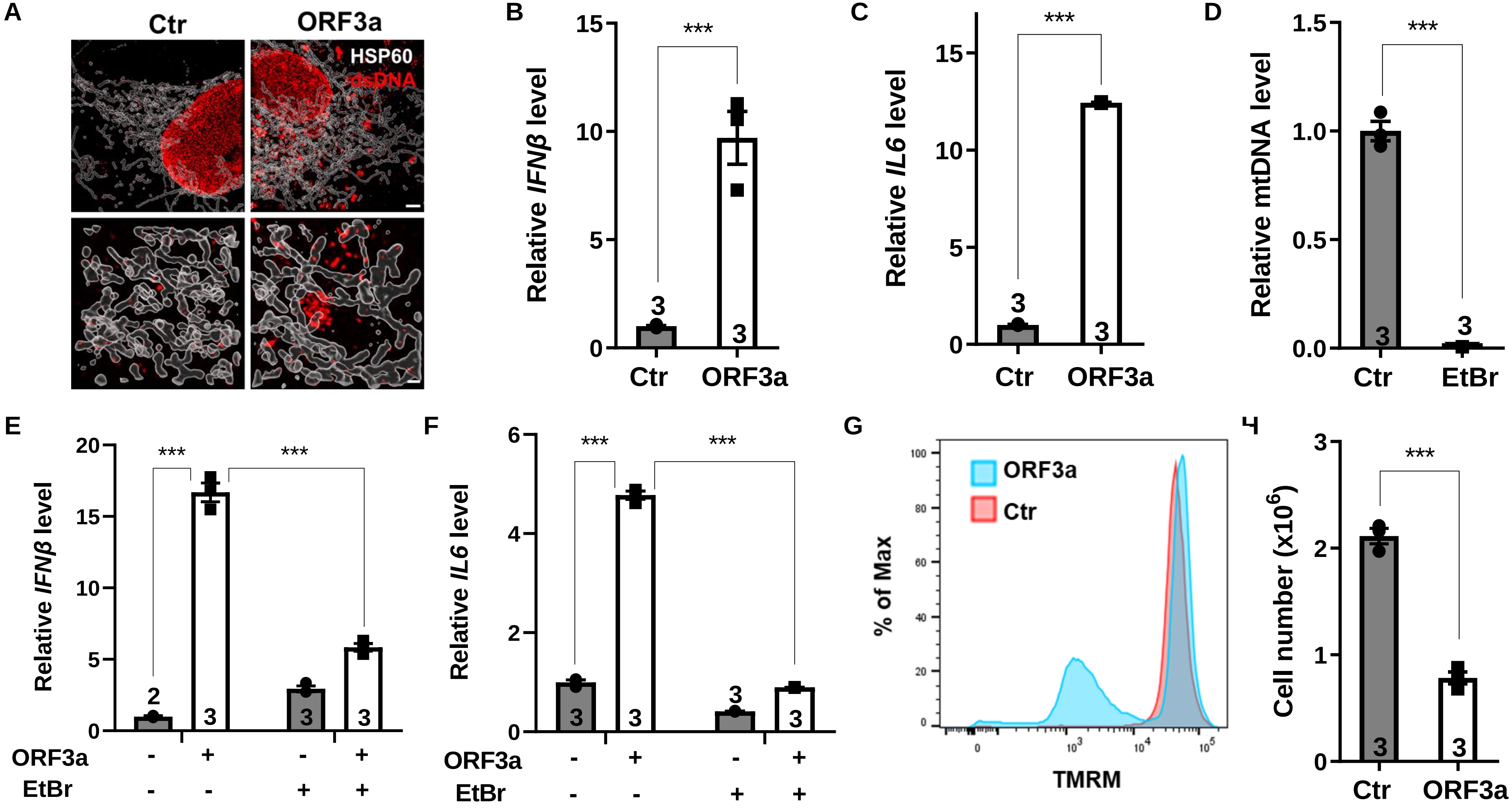

Figure S9. Effects of ORF3a expression in HeLa cells. Related to Figure 5.

A-C, HeLa cells were infected with control or ORF3a-expressing lentivirus.

A, Lattice SIM 3D super-resolution images for immunostaining with anti-DNA (DNA) and anti-HSP60 (mitochondria) antibodies (Scale bar: 3μm (top) and 0.7μm (bottom)).

B, C, Quantitative real-time PCR analyses for the mRNA levels of the indicated genes.

D, Quantitative real-time PCR analyses for the mtDNA levels in HeLa cells treated with or without ethidium bromide (EtBr).

E, F, Quantitative real-time PCR analyses for the mRNA levels of the indicated genes in HeLa cells treated with or without ethidium bromide followed by infection with control or ORF3a-expressing lentivirus.

G, Flow cytometry analysis of HeLa cells infected with control or ORF3a-expressing lentivirus followed by staining with TMRM.

H, Cell number. Data are mean ± s.e.m. *** $p < 0.001$. n=2-5 independent experiments.

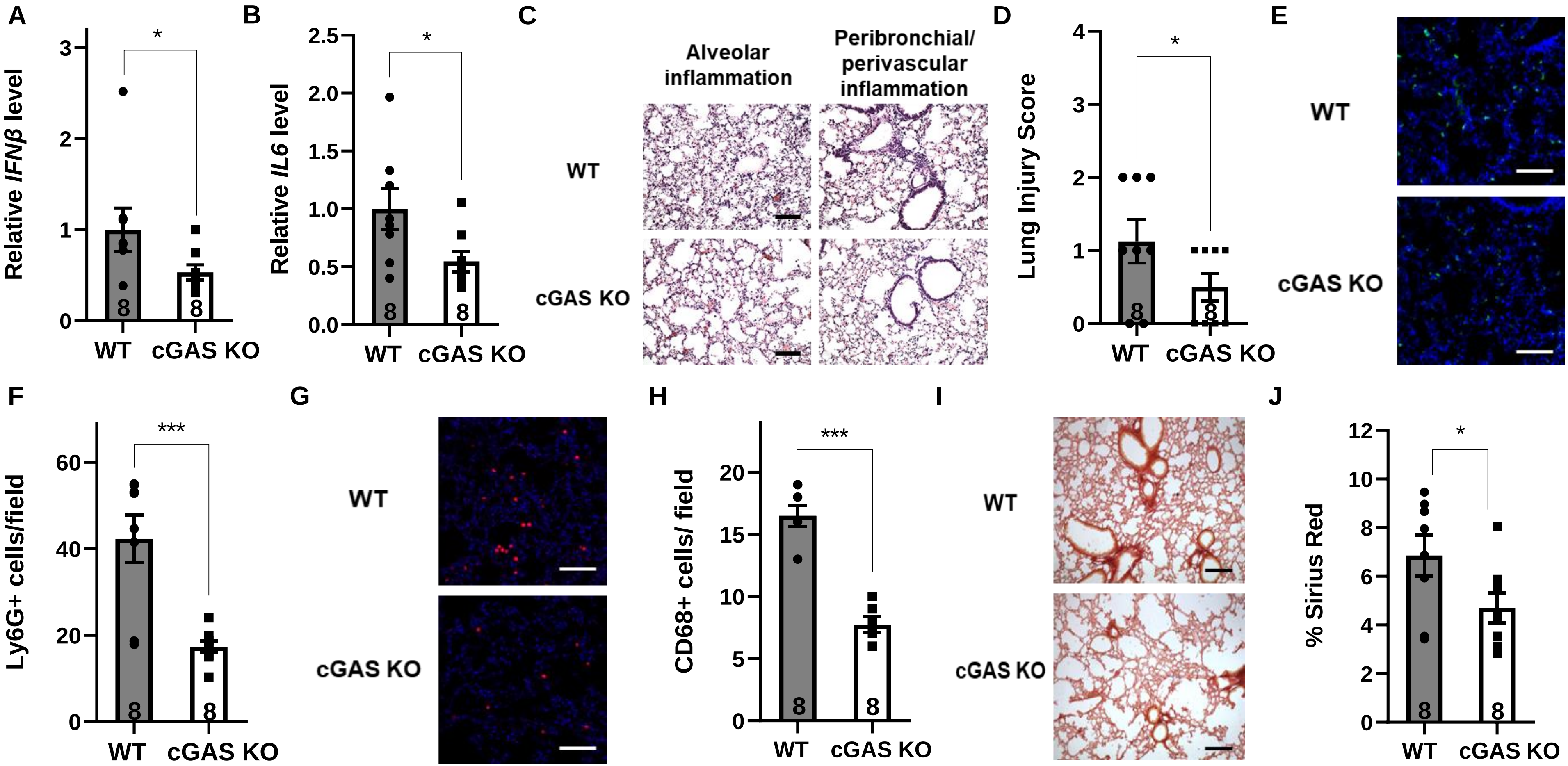

Figure S10. ORF3a triggers lung inflammation and immunopathology via cGAS. **Related to Figure 7.**

WT or cGAS KO mice were inoculated intranasally with ORF3a-expressing AAV6. Mice were analyzed 3 weeks later.

A, B, Quantitative real-time PCR analyses for the mRNA levels of the indicated genes in BALF cells. n=2 independent experiments.

C, D, H&E staining (C) and quantification (D) of lung sections. Scale bar: 100 μm. n=8 mice/group, 10-15 images examined from 3 slides/mouse.

E, F, Immunostaining for Ly6G+ cells (E) and quantification (F) of lung sections. Scale bar: 100 μm. n=8 mice/group, 6 images examined from 3 slides/mouse.

G, H, Immunostaining for CD68+ cells (G) and quantification (H) of lung sections. Scale bar: 100 μm. n=8 mice/group, 4 images examined from 3 slides/mouse.

I, J, Sirius red staining (I) and quantification (J) of lung sections. Scale bar: 200 μm. n=8 mice/group, 10 images examined from 3 slides/mouse. Data are mean ± s.e.m. * $p < 0.05$. *** $p < 0.001$.

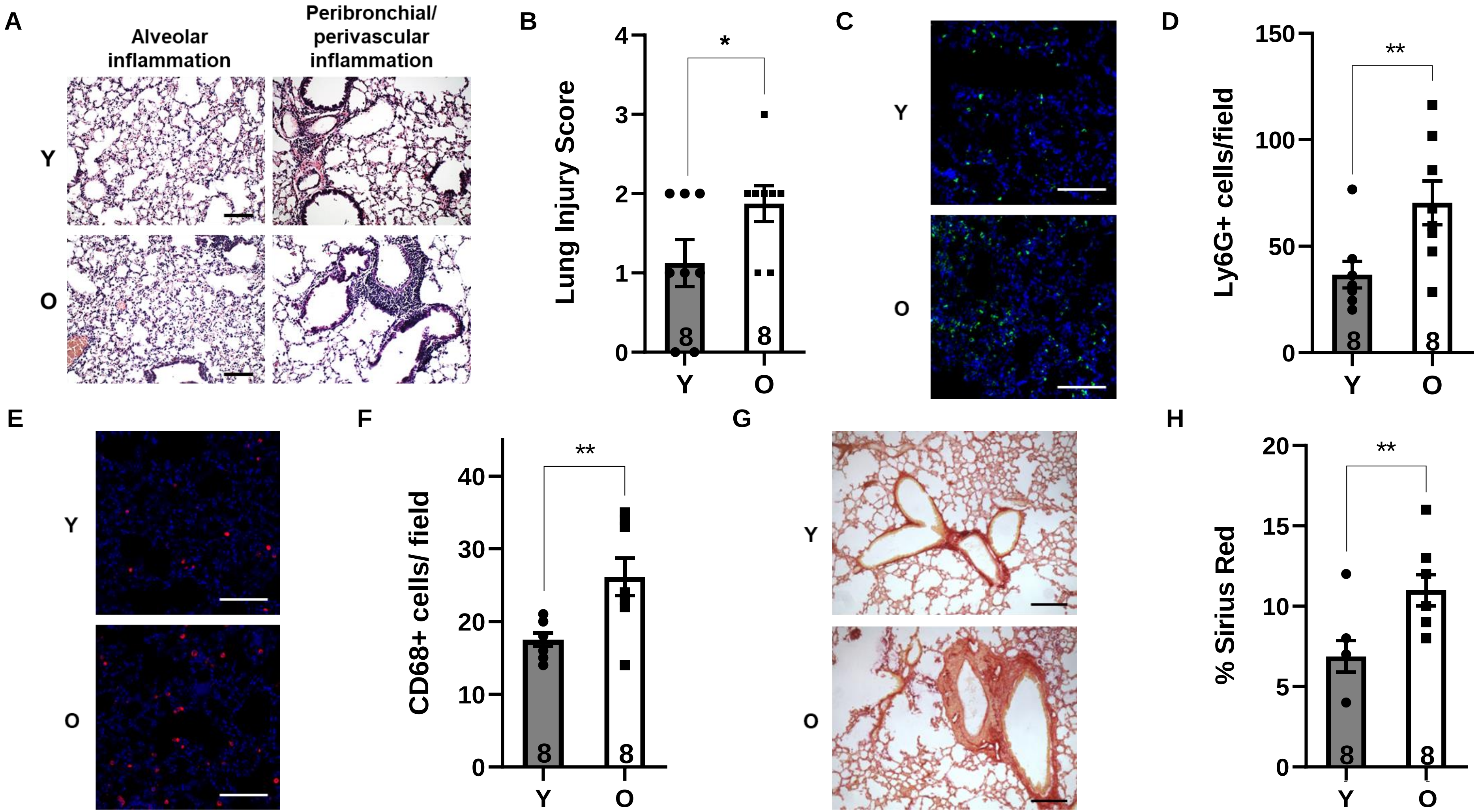

Figure S11. Aging exacerbates ORF3a-induced lung inflammation and immunopathology. Related to Figure 7.
Young (5 months old) or old mice (24 months old) were inoculated intranasally with ORF3a-expressing AAV6. Lung sections were analyzed 3 weeks later.
A, B, H&E staining (A) and quantification (B). Scale bar: 100 μm. n=8 mice/group, 10-15 images examined from 3 slides/mouse.
C, D, Immunostaining for Ly6G+ cells (C) and quantification (D). Scale bar: 100 μm. n=8 mice/group, 6 images examined from 3 slides/mouse.
E, F, Immunostaining for CD68+ cells (E) and quantification (F). Scale bar: 100 μm. n=8 mice/group, 4 images examined from 3 slides/mouse.
G, H, Sirius red staining (G) and quantification (H). Scale bar: 200 μm. n=8 mice/group, 10 images examined from 3 slides/mouse.
Data are mean ± s.e.m. * $p < 0.05$. ** $p < 0.01$.

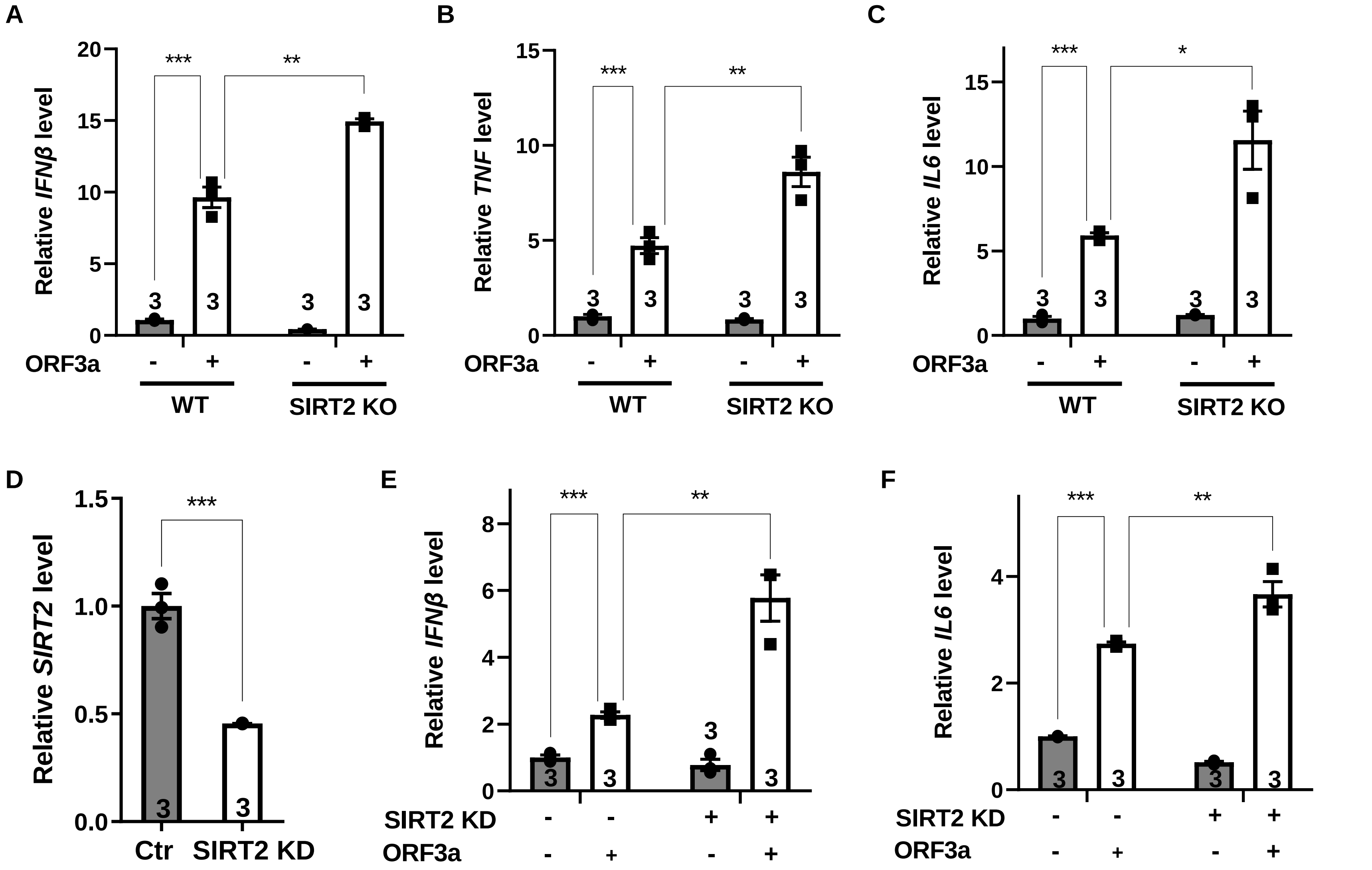

Figure S12. SIRT2 inhibits ORF3a-induced inflammatory response. Related to Figure 7.

A-C, Quantitative real-time PCR analyses for the mRNA levels of the indicated genes in WT or SIRT2 KO MEFs infected with ORF3a-expressing lentivirus.

D-F, Comparison of control and SIRT2 knockdown HeLa cells.

D, Quantitative real-time PCR analyses for the mRNA levels of SIRT2.

E, F, Quantitative real-time PCR analyses for the mRNA levels of the indicated genes in cells infected with control or ORF3a-expressing lentivirus.

Data are mean ± s.e.m. * $p < 0.05$. ** $p < 0.01$. *** $p < 0.001$. n=2 independent experiments.

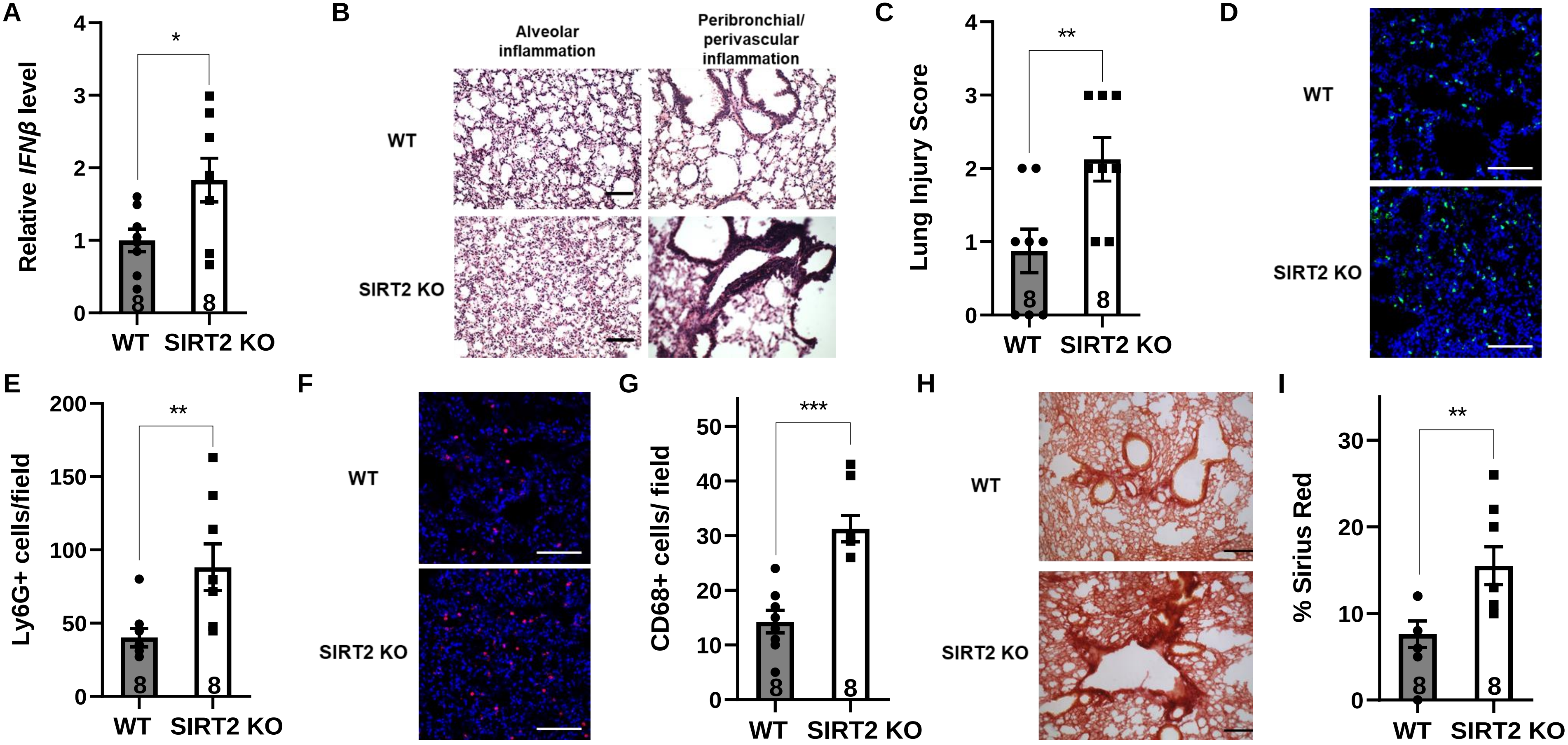

Figure S13. SIRT2 inhibits ORF3a-induced lung immunopathology. Related to Figure 7.

WT or SIRT2 KO mice were inoculated intranasally with ORF3a-expressing AAV6. Mice were analyzed 3 weeks later.

A, Quantitative real-time PCR analyses for the mRNA levels of IFNβ in the lungs. n=2 independent experiments.

B, C, H&E staining (B) and quantification (C) of lung sections. Scale bar: 100 μm. n=8 mice/group, 10-15 images examined from 3 slides/mouse.

D, E, Immunostaining for Ly6G+ cells (D) and quantification (E) of lung sections. Scale bar: 100 μm. n=8 mice/group, 6 images examined from 3 slides/mouse.

F, G, Immunostaining for CD68+ cells (F) and quantification (G) of lung sections. Scale bar: 100 μm. n=8 mice/group, 4 images examined from 3 slides/mouse.

H, I, Sirius red staining (H) and quantification (I) of lung sections. Scale bar: 200 μm. n=8 mice/group, 10 images examined from 3 slides/mouse.

Data are mean ± s.e.m. * $p < 0.05$. ** $p < 0.01$. *** $p < 0.001$.

Table S1. Primers used in qPCR

mGAPDH	Forward	ACC CAG AAG ACT GTG GAT GG
	Reverse	ACA CAT TGG GGG TAG GAA CA
mIFNB1	Forward	CAG CTC CAA GAA AGG ACG AAC
	Reverse	GGC AGT GTA ACT CTT CTG CAT
mTNF	Forward	CAT CTT CTC AAA ATT CGA GTG ACA A
	Reverse	TGG GAG TAG ACA AGG TAC AAC CC
mIL6	Forward	CAA CCA AGA GGT GAG TGC TTC
	Reverse	GGT GTC CTC TTT CCC ACA CTG
mSIRT2	Forward	TGG GCT GGA TGA AAG AGA A
	Reverse	GGT CCA CCT TGG AGA AGT CTG
mND4	Forward	AAC GGA TCC ACA GCC GTA
	Reverse	AGT CCT CGG GCC ATG ATT
mTert	Forward	CTA GCT CAT GTG TCA AGA CCC TCT T
	Reverse	GCC AGC ACG TTT CTC TCG TT
mIfit1	Forward	TCT AAA CAG GGC CTT GCA G
	Reverse	GCA GAG CCC TTT TTG ATA ATG T
mIsg15	Forward	AGC AAT GGC CTG GGA CCT AAA
	Reverse	AGC CGG CAC ACC AAT CTT
hGAPDH	Forward	GAG TCA ACG GAT TTG GTC GT
	Reverse	TTG ATT TTG GAG GGA TCT CG
hIFNB1	Forward	GCT TGG ATT CCT ACA AAG AAG CA
	Reverse	ATA GAT GGT CAA TGC GGC GTC
hTNF	Forward	TGG CCC AGG CAG TCA GA
	Reverse	GGT TTG CTA CAA CAT GGG CTA CA
hIL6	Forward	ACT CAC CTC TTC AGA ACG AAT TG
	Reverse	CCA TCT TTG GAA GGT TCA GGT TG
hSIRT2	Forward	CAG GAG GCT CAG GAC TCA GA
	Reverse	GGC TGA GCG TCT GGG AGA
hND1	Forward	CCC TAA AAC CCG CCA CAT CT
	Reverse	GAG CGA TGG TGA GAG CTA AGG T
hβ-actin	Forward	CTG GAA CGG TGA AGG TGA CA
	Reverse	AAG GGA CTT CCT GTA ACA ATG CA
cGAS	Forward	GAG GCG CGG AAA GTC GTA A
	Reverse	TTG TCC GGT TCC TTC CTG GA
